# *Celf4* Regulates Excitability of Bushy Cells in the Cochlear Nucleus of the Mouse Brainstem

**DOI:** 10.1007/s10571-026-01732-8

**Published:** 2026-04-24

**Authors:** Yige Li, Siyu Qiu, Fang Wang, Zhenghong Bi, Zhiyong Liu, Yingzi He, Geng-Lin Li

**Affiliations:** 1 Department of Otorhinolaryngology, ENT Institute, and NHC Key Laboratory of Hearing Medicine, Eye & ENT Hospital, Fudan University, 83 Fenyang Rd, Xuhui District, Shanghai, 200031 China; 2 Shanghai Key Laboratory of Gene Editing and Cell Therapy for Rare Diseases, Fudan University, Shanghai, 200031 China; 3 Institutes of Brain Science, State Key Laboratory of Medical Neurobiology, and MOE Frontiers Center for Brain Science, Fudan University, Shanghai, 200031 China; 4 Frontier Interdisciplinary Domain, Beijing Institute of Technology, Zhuhai, 519088 China; 5 Institute of Neuroscience, State Key Laboratory of Neuroscience, CAS Center for Excellence in Brain Science and Intelligence Technology, Chinese Academy of Sciences, Shanghai, 200031 China; 6 University of Chinese Academy of Sciences, Beijing, 101408 China

**Keywords:** *Celf4*, Ribbon synapse, Endbulb of held synapse, Synaptic transmission, Intrinsic excitability, 18q12.2 deletion syndrome

## Abstract

The CUGBP Elav-like family 4 (CELF4), an RNA-binding protein, is dynamically expressed in spiral ganglion neurons (SGNs) in the cochlea over development, but how *Celf4* is involved in regulating hearing functions is poorly understood. In this study, we generated a *Celf4*^±^ mouse line and examined changes in the first two synapses along the auditory pathways. Firstly, we found that hearing functions were largely intact in *Celf4*^±^ mice, with exception of reduced amplitude for Wave II of auditory brainstem responses (ABRs) and increased delays for Wave II and IV, both in case of 4 kHz only. Secondly, we found that counts of inner and outer hair cells (IHCs and OHCs) and SGNs remained unchanged in *Celf4*^±^ mice, and that the number and function of ribbon synapses between IHCs and SGNs were comparable between WT and *Celf4*^±^ mice. Lastly, function of the endbulb of Held synapse, formed between auditory nerve fibers (ANFs) of SGNs and bushy cells in the cochlear nucleus, was significantly altered in *Celf4*^±^ mice. Specifically, synaptic vesicle release was subtly reduced, and excitability of bushy cells was significantly dampened, likely due to a hyperpolarized resting membrane potential. Furthermore, we found that spike kinetics was significantly faster in *Celf4*^±^ bushy cells, likely caused by a larger fast-inactivating A-type potassium current (I_A_) found in these cells. In conclusion, we found that *Celf4* haploinsufficiency altered transmission at the endbulb of Held synapses in the cochlear nucleus in a significant and multifaceted manner, revealing the roles of *Celf4* in regulating hearing functions.

**Graphical Abstract:**

Title: Schematic representation of the experimental approach and major findings.

Legend: In mice with *Celf4* haploinsufficiency, we found that while function at hair cell ribbon synapses was largely preserved, transmission at the endbulb of Held synapse was significantly altered, including subtly reduced release of synaptic vesicles from the endbulb, dampened excitability in bushy cells, and accelerated spike kinetics caused by enhanced voltage-gated K^+^ current.

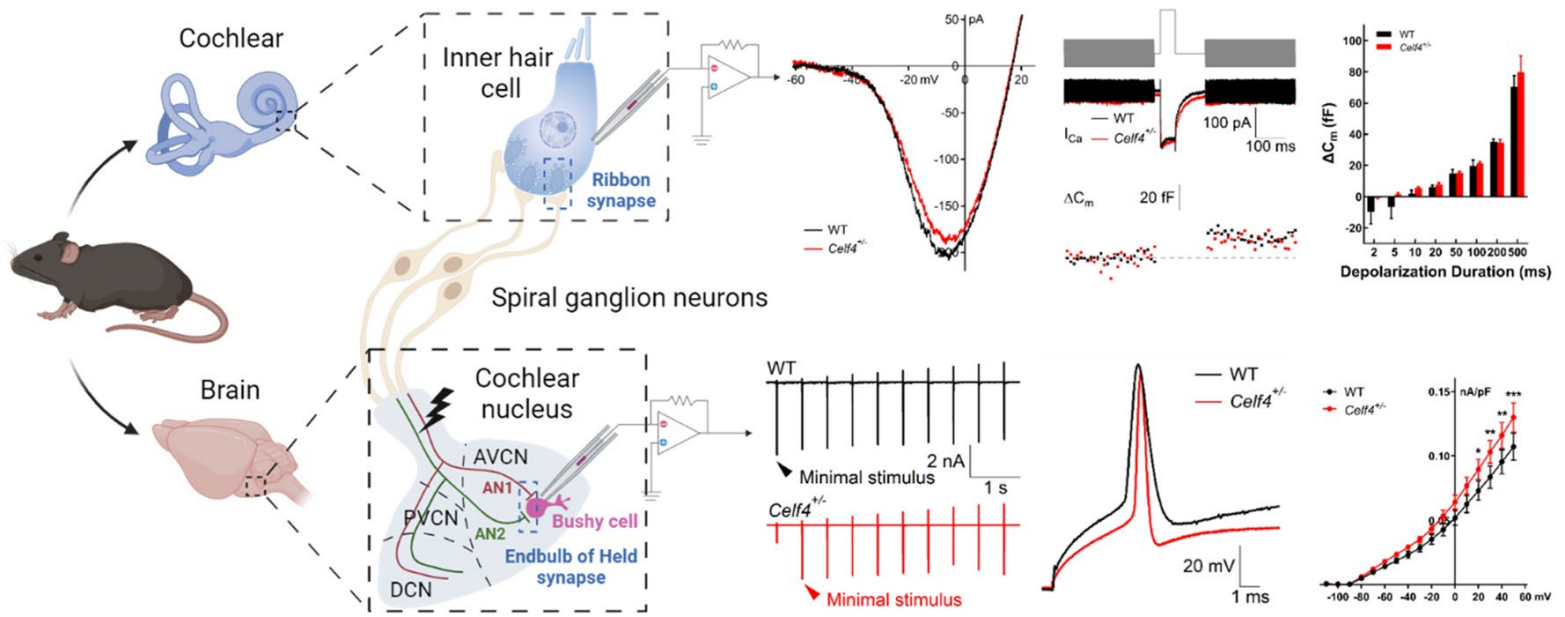

**Supplementary Information:**

The online version contains supplementary material available at 10.1007/s10571-026-01732-8.

## Introduction

Gene expression in the brain is highly dynamic not only spatially but also temporally, and posttranscriptional processing provides an important layer for regulating gene expression, mainly through interdependent networks of RNA-binding proteins (Schieweck et al. 2021). Among them, the CUGBP Elav-like family (CELF, 1–6) that binds to CUG repeats is highly conserved across different species and dysfunction of CELF is implicated in various neurological disorders. In particular, deficiency for CELF4 in mice leads to both convulsive and nonconvulsive epilepsy, depending on gene dosage and genetic background. Interestingly, the timing of CELF4 disruption also affects the seizure phenotype, with removal of CELF4 within first postnatal week exhibiting absence-like non-convulsive seizures, while knockout of CELF4 in adulthood leading to convulsive seizures (Yang et al. 2007; Wagnon et al. 2011). Furthermore, loss of CELF4 results in depression-like behaviors due to alteration in formation of dendritic spines that affects communication among pyramidal neurons (Shen et al. 2022). In humans, haploinsufficiency of CELF4, which occurs when a single gene copy fails to produce enough protein for normal function, is implicated in 18q12.2 deletion syndrome phenotypes, including seizures, intellectual disabilities, and other behavioral disorders (Halgren et al. 2012). Further clinical studies suggest that CELF4 haploinsufficiency also leads to developmental delays, motor coordination disorders, emotional disturbances, neuroticism, and autism or autistic-like behaviors (Barone et al. 2017; An et al. 2019). In addition, numerous case studies support the robust association between heterozygous CELF4 variants and neurodevelopmental disorders including speech and language delay, anxiety and ADHD (Bruel et al. 2025).

On the molecular level, lacking of CELF4 significantly downregulates four genes, including *Htr2c*, *Syn-2*, *Nsf* and *Snca*, all of which are related to seizures and synaptic transmission (Yang et al. 2007). In transcriptome analysis, mRNAs bound to CELF4 accounts for 15%-20% of all mRNAs and they are highly enriched for functions in synaptic plasticity and neurotransmission (Wagnon et al. 2012). CELF4 in the neocortex is enriched in both the marginal region and the subplate for initial synaptogenesis, and binds to synapse-associated mRNAs including *Atp6v0c*, *Sv2a*, *Syn2* and *Gabra3*. During prenatal synaptogenesis of neocortex, several synaptic mRNAs and CELF4 targets, including *Map1b*, *Ppp1r9a*, *Sv2a*, *Syp*, and *vGlut1*, are significantly changed after CELF4 deletion. Notably, the mRNAs bound to CELF4 are encoded by risk genes threatening neurodevelopment and translate into synaptic proteins (Salamon et al. 2023). On the functional level, CELF4 is expressed predominantly in excitatory neurons in cerebral cortex, hippocampus, striatum and reticular thalamus, and mostly responsible for excitatory synaptic transmission. Disruption of CELF4 in mice increases the frequency and amplitude of miniature excitatory postsynaptic currents (mEPSCs), and hyperactivating the synaptic transmission (Wagnon et al. 2011). In addition, whether CELF4 is null or heterozygous, the overexpressed sodium channel Na_V_1.6 protein, encoded by *Scn8a* mRNA that directly binds CELF4, at the axon initial segment of cortical pyramidal neurons, enhances neuronal excitability by lowering threshold for action potential generation, enhancing persistent I_Na_, and hyperpolarizing its half-activation potential (Sun et al. 2013).

Besides the central nervous system, CELF4 is also expressed in the sensory systems. Reportedly, CELF4 is expressed in the dorsal root ganglia neurons that respond to specific stimuli such as light touch, temperature and stretch (Grlickova-Duzevik et al. 2023). Also, CELF4 is enriched in the initial axon of retinal ganglion cells during embryonic development, then appears in amacrine and bipolar cells, mostly locating at the synaptic boutons of rod bipolar cells at postnatal day 14 (P14) (Karunakaran et al. 2013). In the auditory pathways, CELF4 has been identified as an SGN-selective gene by bulk RNA-Seq analysis and validated by the generation of a *Celf4* knock-in mouse strain. Moreover, CELF4 has been shown to dynamically express in SGNs in both embryonic and adult period (Li et al. 2020). Besides the periphery, protein expression data from the Human Protein Atlas indicate that CELF4 is also present in multiple central auditory nuclei, including the cochlear nucleus, superior olivary complex, inferior colliculus, medial geniculate body, and auditory cortex. Although function of CELF4 in brain regions of high-order functions has been extensively studied, its function in sensory synapses remains poorly understood. To address this question, we generated a mouse *Celf4*^±^ line where *Celf4* was genetically knocked out in half and investigated functions of *Celf4* in regulating hearing. Firstly, we recorded ABRs and distortion product otoacoustic emissions (DPOAEs) of both wild-type and *Celf4*^±^ mice and evaluated their hearing performance. Secondly, we conducted patch-clamp recording in whole-mounted cochleae and investigated functions of hair cell ribbon synapses. Lastly, we performed patch-clamp recording in brainstem slices and examined the excitability of bushy cells in the cochlear nucleus and synaptic transmission in the endbulb of Held synapse.

## Materials and Methods

### Animals and Hearing Assessments In Vivo

The *Celf4* knockout mouse line (*Celf4*^±^) was generated by co-injecting Cas9 protein, a pair of sgRNAs targeting exon 3 and exon 12, and a 120 bp single-stranded DNA donor into fertilized eggs from wild-type C57BL/6 J mice. The successful knockout was confirmed by PCR genotyping using two primer sets: one set amplifies a 669 bp fragment for both genotypes, and the other set is specific to the knockout allele (See in Table S3). The mouse colony was maintained by crossing *Celf4*^±^ mice with *Celf4*^+*/*+^ mice. Wild-type (WT) littermates from these crosses were used as controls throughout the study. The mice were bred in an animal facility in Fudan University with standard food, water, and 12/12-h light/dark cycle. WT and *Celf4*^±^ mice of both sex with an age around 4 weeks were used for all experiments, except for recording of auditory brainstem responses (ABRs) where experiments were performed additionally in mice of 10 months, to examine possible progressive hearing loss in *Celf4*^±^ mice.

ABR measurements were conducted in a soundproof chamber. The free-field sound stimuli were delivered through a speaker (Tucker-Davis Technologies, Inc., Alachua, FL, USA) placed in front of the anesthetized animal 10 cm away from the vertex. Digitized sound signals were generated through an acoustic stimulation system (Tucker-Davis Technologies), and data were acquired with the software BioSigRZ (Tucker-Davis Technologies). Stimulus frequencies ranged from 32 to 4 kHz in half-octave steps and sound intensity started from 90 dB SPL, descending in 5 dB steps to 20 dB SPL. Tone pips (3 ms duration, 0.5 ms rise/fall time) were used at various frequencies. ABRs were recorded via three subdermal needle electrodes: the vertex of the skull (recording), the mastoid area of the right ear (reference) and the rump of the left side (ground). For each sound stimulus, a total of 300 responses were collected and averaged, and the ABR threshold defined as the lowest sound intensity that evoked a visible and reproducible waveform. The amplitudes and latencies of the ABR Wave I-IV were measured and computed manually offline.

For each experiment, DPOAEs were elicited by two pure tones of frequencies (ƒ_1_ and ƒ_2_), which were generated by a Tucker-Davis Technologies (TDT) RZ6 system driving two speakers (TDT) via separate channels. To produce 2ƒ_1_ − ƒ_2_ DPOAEs, two equal level (L_1_ = L_2_) stimulus frequencies (ƒ_1_ and ƒ_2_) with a constant ƒ_2_/ƒ_1_ ratio of 1.2 were used to generate DP-grams. ƒ_1_ and ƒ_2_ are presented at the same time, sweeping ƒ_2_ as 8.7, 17.4 and 34.8 kHz, and the sound level was decreased from 80 to 20 dB SPL in 5 dB steps. The I–O function was analyzed to account for the amplitude of the DPOAE at each frequency, background noise levels were determined by the average of five points before and after the point at 2ƒ_1_ − ƒ_2_, and DPOAE thresholds were measured as the lowest sound intensity that generated a DPOAE that is 6 dB higher in amplitude than the background noise level. All amplitudes of 2ƒ_1_ − ƒ_2_ and noise floor levels were analyzed by using BioSigRZ software (TDT).

### Patch-Clamp Recording

For whole-mount cochlear recordings, the apical turn was freshly isolated and placed in an oxygenated extracellular solution with the following composition (in mM): 123 NaCl, 10 D-glucose, 10 HEPES, 5.8 KCl, 5 CaCl_2_, 2 sodium pyruvate, 0.9 MgCl_2_ and 0.7 NaH_2_PO_4_ (pH 7.35, 300 mOsm). The tissue was visualized using an up-right microscope (Olympus) equipped with a 61 × water-immersion objective. Patch-clamp recordings were performed using an EPC10/2 amplifier (HEKA Electronics, Lambrecht Pfalz, Germany) controlled by Patchmaster software (HEKA Electronics). Borosilicate glass capillaries (Sutter) were used to fabricate recording pipettes, which were filled with an internal solution containing (in mM): 135 Cs-methane, 10 CsCl, 10 TEA-Cl, 10 HEPES, 3 Mg-ATP, 2 EGTA, and 0.5 Na-GTP (pH 7.25, 290 mOsm). Cells were voltage-clamped at a holding potential of −90 mV. To record I_Ca_ in IHCs, a voltage ramp from −90 to 70 mV was applied. The resulting current–voltage relationship (I–V curve) was fitted using a Boltzmann equation to derive V_half_ and κ:

$$\text{I }\left(\mathrm{V}\right)=(\mathrm{V}-{\mathrm{V}}_{\mathrm{rev}}) \times \frac{{\mathrm{G}}_{\mathrm{max}}}{1+\mathrm{exp}(-\frac{\mathrm{V}-{\mathrm{V}}_{\mathrm{half}}}{\upkappa })}$$

where V_half_ is the half activation potential, κ is the slope factor, V_rev_ is the reversal potential and G_max_ is the maximum chord conductance.

For capacitance measurements, exocytosis was induced by depolarizing the cell to −10 mV for durations ranging from 2 to 500 ms. The holding potential was modulated by a 1 kHz sine wave (50 mV peak-to-peak) applied before and after depolarization (Li et al. 2009). Whole-cell capacitance changes were calculated from the resulting current responses using the “LockIn” feature in conjunction with the “Sine + DC” method (Lindau and Neher 1988; Gillis 2000). The capacitance increase (ΔC_m_) in individual IHCs was plotted against the stimulation duration (t) and fitted using a combination of an exponential function for readily releasable pool (RRP) of synaptic vesicles (C_m,RRP_) and depletion time of RRP (τ_RRP_), and a linear function for sustained release rate (SRR, R_sustained_):

$$\Delta {\mathrm{C}}_{\mathrm{m}} \left(\mathrm{t}\right)={\mathrm{C}}_{\mathrm{m},\mathrm{RRP}} \times \left(1 -\text{ exp }\left(\frac{\mathrm{t}}{{\uptau }_{\mathrm{RRP}}}\right)\right) + {\mathrm{R}}_{\mathrm{sustained}} \times \text{ t}$$

The values in capacitance changes were converted to number of synaptic vesicles with a ratio of 37 aF per synaptic vesicle (Lenzi et al. 1999; Johnson et al. 2009).

Sagittal brainstem slices (200–250 μm thick) containing the anteroventral cochlear nucleus (AVCN) were prepared using a vibratome (Leica, VT1200 S) in an ice-cold slicing solution composed of (in mM): 230 sucrose, 25 NaHCO_3_, 10 D-glucose, 3 MgCl_2_, 3 myo-inositol, 2.5 KCl, 2 sodium pyruvate, 1.25 NaH_2_PO_4_, 0.4 sodium L-ascorbate and 0.1 CaCl_2_, which was continuously bubbled with mixed oxygen (95% O_2_ and 5% CO_2_). The slices were then collected and incubated at 34 °C for at least 40 min in mix-oxygenated artificial cerebrospinal fluid (ACSF) solution containing (in mM): 125 NaCl, 25 NaHCO_3_, 10 d-glucose, 3 myo-inositol, 2.5 KCl, 2 sodium pyruvate, 1.8 MgCl_2_, 1.25 NaH_2_PO_4_, 1.2 CaCl_2_ and 0.4 sodium l-ascorbate. For patch-clamp recordings, single AVCN slice was transferred to a submersion chamber and perfused with recirculating, oxygenated ACSF at room temperature (RT). Two distinct intracellular solutions were employed: one for recording spontaneous and evoked EPSCs containing the following (in mM) 130 Cs-methanesulfonate, 10 CsCl, 10 Na_2_-phosphocreatine, 10 HEPES, 5 EGTA, 4 Mg-ATP, 3 QX314-Cl and 0.3 Na_2_-GTP (pH 7.3, 290 mOsm); the other for recording spike containing the following (in mM) 130 K-gluconate, 10 KCl, 10 HEPES, 10 creatine phosphate, 3 Mg-ATP, 2 EGTA and 0.5 Na_2_-GTP (pH 7.3, 290 mOsm). For isolation of A-type (rapidly inactivating) potassium current, recordings were performed with QX314-Cl (3 mM) included in the internal solution to suppress sodium current, and TEA-Cl (10 mM) was applied in the bath. For isolation of sodium current, recordings were performed with internal solution containing Cs^+^, and TEA-Cl (10 mM) and 4-AP (2 mM) was applied in the bath. Evoked EPSCs were elicited by stimulating the auditory nerve with brief voltage pulses (1–10 V, 0.1 ms duration) generated by a Master-9 Pulse Stimulator (AMPI, Israel) and delivered via a suction pipette. Bushy cells were identified based on established electrophysiological signatures, including single spike firing in response to prolonged current injection under current-clamp (Wu and Oertel 1984; Xie 2016; Antunes et al. 2020), and synaptic currents with a large amplitude (> 2 nA) under voltage-clamp. Series resistance was typically between 5 and 20 MΩ, uncompensated. To assess axonal excitability, we applied a series of near-threshold stimuli (increment of 0.2 V) to the auditory nerve and recorded eEPSCs in bushy cells. For each cell, we delivered multiple trials at each stimulation voltage and calculated the stimulation success rate as the proportion of trials that elicited a measurable eEPSC. We then plotted success rate against stimulation voltage for each cell and fitted the data with a Boltzmann function to derive the half activation voltage (V_half_) for evaluating the excitability of auditory nerve fibers. To examine the dynamics of synaptic vesicle release, we evoked 50 EPSCs at 100 Hz, and calculated the sustained release rate as the average quantal content of the last 20 EPSCs divided by stimulation interval (i.e., 10 ms for 100 Hz). We then calculated the readily releasable pool of synaptic vesicles by subtracting 50 times of the average quantal content of the last 20 EPSCs from the total quantal contents for all 50 EPSCs. Total membrane currents were recorded by applying voltage steps of 100 ms from −90 to 100 mV in 10 mV increments from a holding potential of −90 mV. To assess spiking behavior, resting membrane potential (RMP) were recorded by holding the cell at absolute zero current, and spikes were then elicited by injecting a series of current steps from both a hyperpolarized potential of −90 mV and the identified RMP.

### Immunofluorescence

Cochleae were isolated and transferred to 4% paraformaldehyde (PFA) at 4 °C overnight for whole-mount staining, and then were decalcified in 120 mM EDTA for 3–4 days, after which the basilar membrane was micro-dissected into apical, middle, and basal turns. For SGNs staining, decalcified cochleae were dehydrated by sequential incubation in 15 and 30% sucrose solutions (each overnight at 4 °C). After embedded in OCT, cochleae were sectioned transversally at a thickness of 20 μm on a sliding microtome. For bushy cells staining, mice were anesthetized and perfused with 20 mL PBS and 20 mL 4% PFA. The whole brain was then removed and post-fixed in 4% PFA overnight at 4 °C. Subsequently, the brain was dehydrated by immersion in 30% sucrose solution for 3 days at 4 °C. Following OCT embedding, brain was coronally sectioned on a sliding microtome to a thickness of 30 μm. The free-floating sections were washed in PBS and subjected to antigen retrieval at 75 °C for 30 min. After cooling to RT, sections were blocked for 2 h at RT and then incubated with primary antibodies for 3 days at 4 °C. The primary antibodies included anti-Myosin 7a (1:500, Proteus Biosciences), anti-Prestin (1:500, Santa Cruz), anti- NF (1:500, Abcam), anti-Homer1 (1:500, Abcam), anti-Ctbp2 (1:500, BD Biosciences), anti- Tuj1 (1:500, Abcam), anti-Vglut1 (1:200, Oasis Biofarm), anti-NeuN (1:200, Oasis Biofarm), and anti-CELF4 (1:25, MilliporeSigma). Appropriate secondary antibodies conjugated to Alexa Fluor 488, 568, or 647 were selected to match the host species of the primary antibodies. For phalloidin staining, cochleae were incubated with Alexa Fluor 488 phalloidin (1:500, Thermo Fisher Scientific) for 30 min in the dark. Subsequently, the tissue was counterstained with DAPI (1:1000, Sigma Aldrich) to visualize cellular nuclei.

### Quantitative PCR (qPCR) Analysis

Transcript levels of *Celf4* and ion channel subunits (*Kcna1* [Kv1.1], *Kcna2* [Kv1.2], *Kcna3* [Kv1.3], *Kcna6* [Kv1.6], *Kcnc1* [Kv3.1], *Kcnd2* [Kv4.2] and *Kcnd3* [Kv4.3]) were quantified by qPCR. Briefly, AVCN tissues were dissected and rinsed in PBS, and then homogenized in TRIzol Reagent (15596018, Invitrogen) using zirconium oxide beads under cryogenic grinding conditions. After centrifugation, the aqueous phase was collected, and then was mixed with chloroform. Total RNA was extracted according to the manufacturer’s protocol, involving phase precipitation with isopropanol, wash with 75% ethanol and dissolution in nuclease-free water. RNA concentration and purity were assessed spectrophotometrically, with samples exhibiting A260/A280 ratios of 1.8–2.2 and A260/A230 ratios > 2.0 being used for subsequent steps. Potential genomic DNA contamination was removed using a gDNA Eraser kit (RR047A, Takara Bio). cDNA was then synthesized from total RNA using PrimeScript RT Enzyme. qPCR reactions were performed in triplicate using TB Green Premix Ex Taq II (RR820A, Takara Bio) on a QuantStudio system. The thermal cycling protocol consisted of an initial denaturation at 95 °C for 3 min, followed by 40 cycles of 95 °C for 10 s and 60 °C for 30 s. A melt-curve analysis was conducted to confirm amplification specificity. The relative expression level of each target gene was calculated using the 2 − ΔΔCt method, with GAPDH serving as the internal reference gene for normalization.

### Confocal Imaging

Cochlear morphology was examined using a Leica SP8 confocal fluorescence microscope with 40 × or 63 × oil objectives. Images were acquired at a resolution of 1024 × 1024 pixels and a scanning speed of 600, utilizing appropriate emission filters for Alexa Fluor 488, 568, and 647. Z-stacks were collected with a consistent optical slice thickness of 2.00 µm. All acquired images were processed using LAS X software, and final figures were assembled in Adobe Illustrator. Quantitative analysis of hair cells (HCs) and spiral ganglion neurons (SGNs) were performed according to established anatomical landmarks. For operational purposes during imaging, the cochlear spiral was divided into three consecutive regions based on its visible turns. Starting from the apex, we imaged regions corresponding to approximately the first 180°, the subsequent ~ 180°, and the final ~ 180° of the spiral, which we designated as the apical, middle, and basal turns, respectively. Surviving HCs were defined as myosin 7a-positive cells and their density was quantified as the number of hair cells per 100 μm length of the cochlear sensory epithelium. This length was measured by tracing the curved contour of the organ of Corti along the arch formed by the inner and outer pillar heads, using the segmented line tool in Fiji. Similarly, SGN survival was assessed by counting β-Tubulin III-positive neuronal cell bodies. For quantification, all Tuj1 and DAPI double-positive cells within each Rosenthal’s canal profile were counted, measured its cross-sectional area, and expressed the result as the number of neurons per 10,000 μm^2^. To quantify the number of ribbon synapses per IHC, segments of 100 μm (same as for HC density quantification) were selected along the curved contour of the basilar membrane. For each segment, Ctbp2/Homer1-positive puncta were counted as ribbon synapses, and the number of IHCs was determined by counting nuclei identified by DAPI staining. The average number of ribbon synapses per IHC was then obtained by dividing the total number of puncta by the number of IHCs within the same segment.

### Data Analysis and Statistical Analyses

Data were analyzed with BioSigRP (Tucker-Davis Technologies Inc., Alachua, FL, USA), Image J, Igor (WaveMetrics, USA) and GraphPad Prism (GraphPad, USA). Throughout the study, all data are presented as mean ± SD, except that mean ± SEM was used in plotted figures for clarity. All samples within each group are biologically independent and the sample sizes (n or N) are determined with considerations of statistical power. To minimize subjective bias, animals were randomly assigned to the experimental and control groups, and the investigators were blinded to group allocation and data collection during the experiments. Normality and homogeneity of variances were both assessed prior to statistical comparisons. The Shapiro–Wilk test was used to test for normality, and Levene’s test was used to assess equality of variances. For data that passed both normality and variance homogeneity tests, parametric tests were applied, while for data that failed either test, non-parametric Mann–Whitney *U* tests were employed. For multiple comparisons, statistical significance was assessed by two-way ANOVA test followed by Sidak multiple comparison test, and p < 0.05 was considered statistically significant.

## Results

### Hearing Functions and Cellular Structure of the Cochlea were Largely Intact in Celf4^±^ Mice

**Fig. 1:**
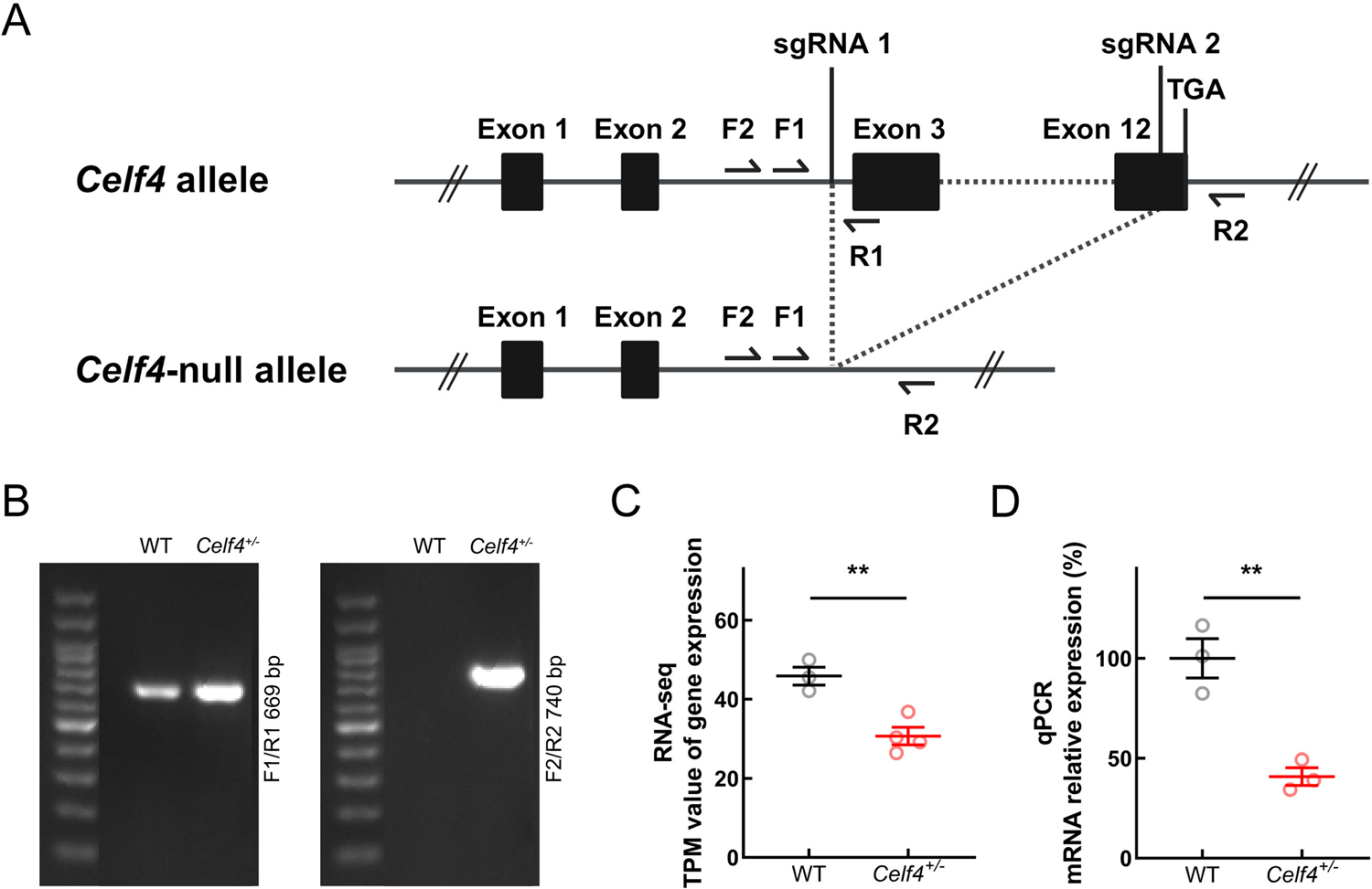
Generation and validation of *Celf4*^±^ mice. **A** Schematic of the CRISPR/Cas9 strategy for disrupting the *Celf4* gene. Two single-guide RNAs (sgRNAs) were designed targeting the 5′ end of exon 3 and the 3′ end of exon 12, respectively. A 120 bp single-stranded DNA donor template consisted of sequences 60 bp upstream of the 5′ sgRNA and 60 bp downstream of the 3′ sgRNA, and was co-injected with Cas9 in fertilized eggs from wild-type C57BL/6 J mice to facilitate a precise deletion from exon 3 to exon 12. **B** Representative genotyping PCR results from a WT and a *Celf4*^±^ mice. The F1/R1 primer pair amplified a 669 bp fragment from both WT and mutant alleles, while the F2/R2 pair yielded a 740 bp product specific to the mutant allele, confirming successful targeting. **C** RNA sequencing (RNA-seq) analysis of AVCN from 4 *Celf4*^±^ mice and 3 WT littermates. Transcripts per million (TPM) values for the *Celf4* gene were significantly reduced in *Celf4*^±^ mice. **D** qPCR validation of AVCN from 3 WT mice and 3 *Celf4*^±^mice. *Celf4* mRNA levels in *Celf4*^±^ mice were approximately 50% of WT levels, confirming haploinsufficiency at the transcriptional level. For this figure and all the other figures, statistical significance was assessed with either *t* test or two-way ANOVA, wherever appropriate, and N.S. indicates p > 0.05 while *, **, *** and **** indicate p < 0.05, 0.01, 0.001 and 0.0001, respectively

**Fig. 2:**
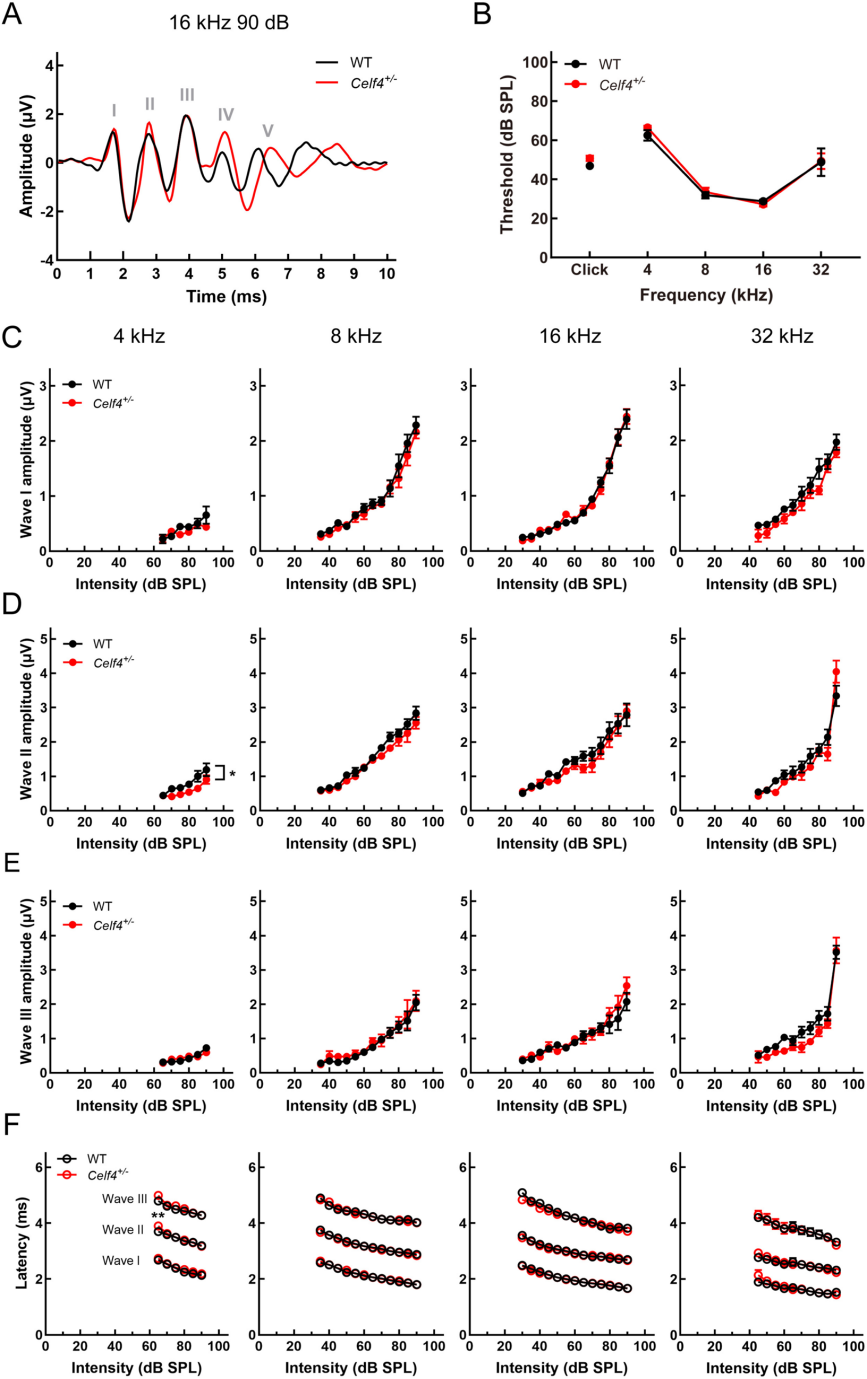
Hearing functions in vivo were largely unchanged in *Celf4*^±^ mice. **A** Representative traces of auditory brainstem response (ABR) recorded from a WT (black) and a *Celf4*^±^ mouse (red), in response to tone burst of 16 kHz and 90 dB SPL. **B** ABR thresholds in WT and *Celf4*^±^ mice, showing no significant difference between the two groups (WT: N = 8 mice, *Celf4*^±^: N = 7 mice). **C**–**F** Amplitudes, **C**–**E** and latencies, **F** for Wave I, II and III in WT and *Celf4*^±^ mice at 4, 8, 16 and 32 kHz, plotted against the sound intensity (WT: N = 8 mice, *Celf4*^±^: N = 7 mice). No significant change was observed in *Celf4*^±^ mice except that Wave II amplitude was significantly reduced (**D**, left) and Wave II latency was significantly increased (**F**, left), both for 4 kHz only

To investigate functional roles of *Celf4* in hearing, we generated a knockout mouse line using CRISPR-Cas9-mediated deletion of exon 3 to exon 12, and the successful removal of the targeted exons was confirmed by genomic sequencing (Fig. 1). Given the low survival rate of homozygotes, we decided to focus on *Celf4*^±^ mice, and employed an interdisciplinary approach to examine functional changes in hearing in these animals. We first conducted ABR and DPOAE recordings and evaluated auditory functions in vivo in WT and *Celf4*^±^ mice of 4 weeks old. We found that WT and *Celf4*^±^ mice had similar ABR waveforms (Fig. 2A and Supplementary Fig. 1A), comparable hearing thresholds (two-way ANOVA: genotype effect: F_(1, 7)_ = 0.812, p = 0.398; interval effect: F_(4, 28)_ = 43.6, p < 0.0001; interaction: F_(4, 23)_ = 0.313, p = 0.866, Fig. 2B), and indistinguishable DPOAE thresholds (two-way ANOVA: genotype effect: F_(1, 7)_ = 0.00775, p = 0.932; interval effect: F_(2, 14)_ = 4.43, p = 0.0323; interaction: F_(2, 14)_ = 1.31, p = 0.302, Supplementary Fig. 2A) and amplitudes (See in Tables S1 and S2, Supplementary Fig. 2C), indicating that hearing functions in whole animals were largely intact. The only exception is at 4 kHz, where the amplitude of Wave II was significantly reduced (two-way ANOVA: genotype effect: F_(1, 7)_ = 7.49, p = 0.0291; interval effect: F_(5, 35)_ = 14.6, p < 0.0001; interaction: F_(5, 26)_ = 0.920, p = 0.484, Fig. 2D) and the latencies of Wave II and IV were significantly increased at 65 dB SPL (See in Tables S1 and S2, Figs. 2F, S1C), whereas, the amplitudes and latencies of the other waves remained normal (See in Tables S1 and S2, Figs. 2C, E and S1B). Notably, at 8 kHz, although the latencies of Wave II and Wave III were shortened in *Celf4*^±^ mice at 10 months, the overall auditory function in *Celf4*^±^ mice was not different from that of wild-type littermates at 1 to 10 months of age (Supplementary Fig. 3), suggesting that hearing with *Celf4*^±^ haploinsufficiency was largely unchanged in long term.

**Fig. 3:**
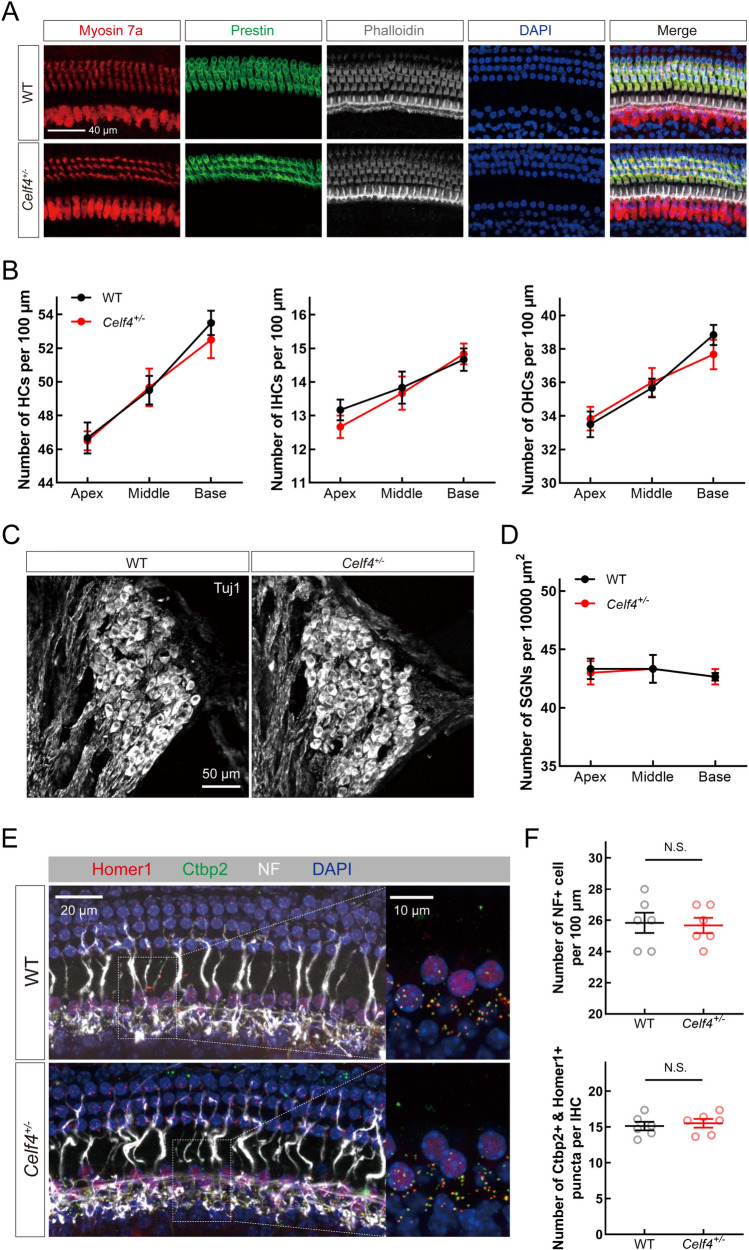
Counts of hair cells, spiral ganglion neurons and ribbon synapses were not changed in the cochlea of *Celf4*^±^ mice. **A** Representative images of whole-mount cochleae from a WT and a *Celf4*^±^ mouse, in the apical turn, quadruple immunolabeled for Myosin7a (hair cells, red), Prestin (outer hair cells, green), Phalloidin (hair bundles, white) and DAPI (nuclei, blue). Scale bar: 40 μm. **B** Counts of all hair cells (HCs, left), inner hair cells (IHCs, middle) and outer hair cells (OHCs, right) in WT (N = 3 mice) and *Celf4*^±^ mice (N = 3 mice), showing that no significant difference was found in all the three turns between the two groups. **C** Representative images of cochleae transversal section from a WT and a *Celf4*^±^ mouse, in the middle turn, immunolabeled with Tuj1 (spiral ganglion neurons, SGNs, white). Scale bar: 50 μm. **D** Counts of SGNs in WT (N = 3 mice) and *Celf4*^±^ groups (N = 3 mice). No significant change was observed in the cochlea of *Celf4*^±^ mice for all the three turns. **E** Representative images of whole-mount cochleae from a WT and a *Celf4*^±^ mouse, in the middle turn, immunolabeled for Homer1 (postsynaptic puncta, red), Ctbp2 (presynaptic puncta, green), NF (neurofilament, white) and DAPI (nuclei, blue). Scale bars: 20 μm (left) and 10 μm (right). **F** Counts of ribbon synapses (colocalized puncta of Homer1 and Ctbp2, bottom) and NF-positive filaments from z-stacks of confocal micrographs, collected from WT (N = 3) and *Celf4*^±^ mice (N = 3). No significant difference was found between the two groups

We then performed immunolabeling in both whole-mount cochleae and cochlear transversal sections of WT and *Celf4*^±^ mice, and we found no significant change in count of hair cells (two-way ANOVA: genotype effect: F_(1, 5)_ = 0.750, p = 0.426; interval effect: F_(2, 10)_ = 48.3, p < 0.0001; interaction: F_(2, 10)_ = 0.257, p = 0.778, Figs. 3A, B and S4) or spiral ganglion neurons (SGNs) (two-way ANOVA: genotype effect: F_(1, 2)_ = 0.0149, p = 0.914; interval effect: F = 0.634, p = 0.577; interaction: F = 0.0124, p = 0.988, Fig. 3C, D, and S5), suggesting that the cellular structure of the cochlea was intact in *Celf4*^±^ mice. We also found no significant change in the numbers of ribbon synapses and neurofilament (NF)-positive cells in *Celf4*^±^ cochleae (WT: number of ribbon synapses = 15.1 ± 1.46, number of NF-positive cells = 25.8 ± 1.60, n = 3 mice; *Celf4*^±^: number of ribbon synapses = 15.5 ± 1.47, number of NF-positive cells = 25.7 ± 1.21, n = 3 mice; number of NF-positive cells: two-tailed unpaired *t*-test, t_(df = 10)_ = 0.203, p = 0.843, number of ribbon synapses: two-tailed unpaired *t*-test, t_(df = 38)_ = 0.705, p = 0.485, Fig. 3E and F).

### Synaptic Functions in Inner Hair Cells in the Cochlea were Intact in Celf4^±^ Mice

**Fig. 4:**
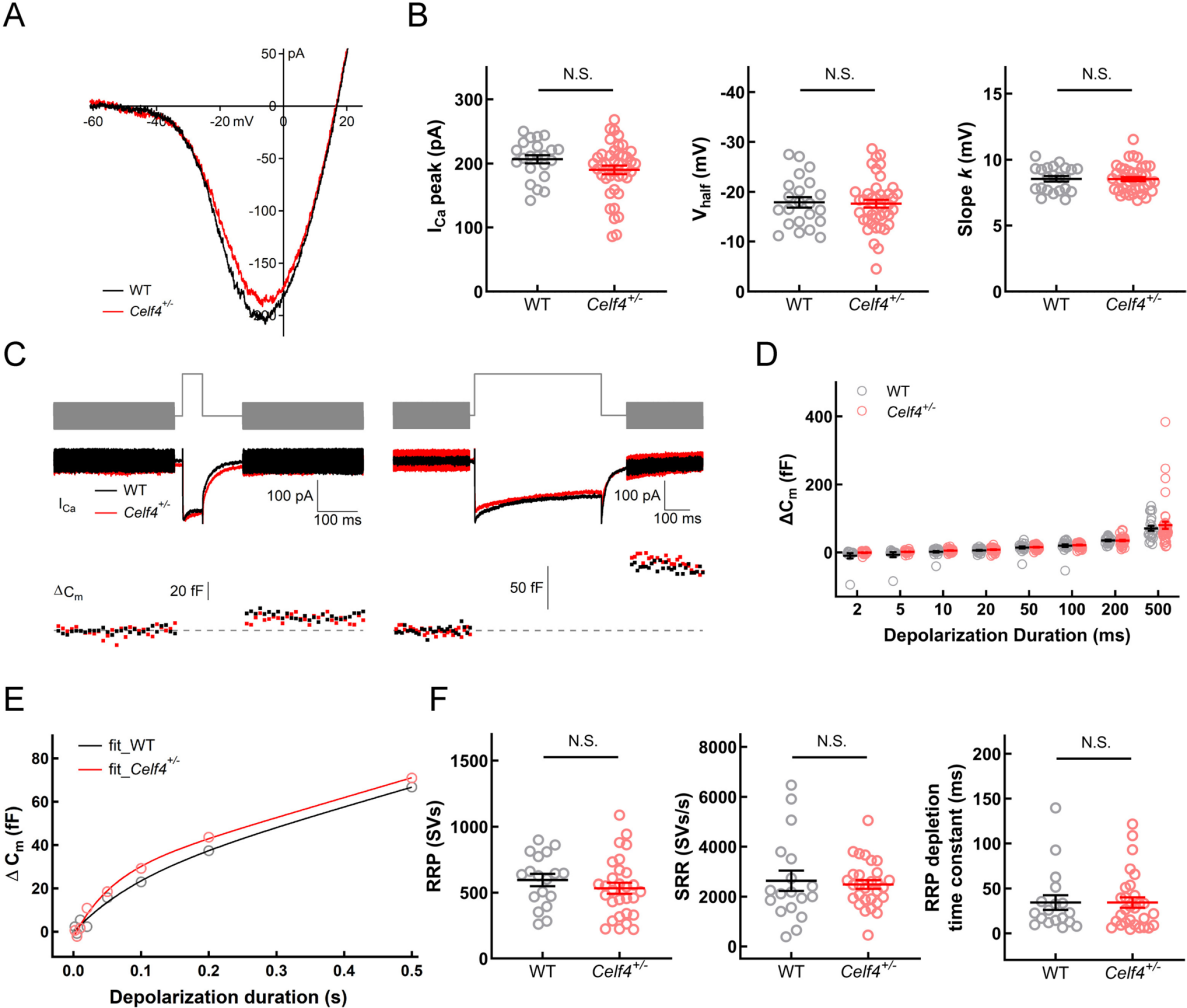
Functions of ribbon synapses in IHCs remained intact in *Celf4*^±^ mice. **A** Representative traces of Ca^2+^ current (I_Ca_) from a WT and a *Celf4*^±^ IHC, induced by voltage ramps, showing that I_Ca_ was not changed in the *Celf4*^±^ IHC. **B** Pooled data on the peak amplitude of Ca^2+^ current (I_Ca_ peak, left), the half-activation voltage (V_half_, middle) and the activation slope (Slope *k*, right) between WT (N = 8 mice, n = 22 cells) and *Celf4*^±^ IHCs (N = 15 mice, n = 41 cells), none of which was changed in *Celf4*^±^ IHCs. **C** Representative I_Ca_ and whole-cell capacitance measurements in a WT and a *Celf4*^±^ IHC. Exocytosis was induced with step depolarization of 50 (left) and 500 ms (right). **D** Pooled data on the capacitance change (ΔC_m_), showing that exocytosis in *Celf4*^±^ IHCs was not changed. **E** and **F** Typical curve fitting of ΔC_m_ over depolarization duration for a WT and a *Celf4*^±^ IHC (**E**), yielding parameters for exocytosis kinetics (**F**), including the readily releasable pool of synaptic vesicles (RRP, left), the sustained release rate of synaptic vesicles (SRR, middle), and the RRP depletion time constant (right). None of the three parameters was changed in *Celf4*^±^ IHCs (WT: N = 8 mice and n = 18 cells, *Celf4*^±^: N = 15 mice and n = 29 cells)

Although hearing functions in vivo were largely intact in *Celf4*^±^ mice, it was still possible that *Celf4* haploinsufficiency caused functional changes at the cellular level, but these changes have opposing effects on hearing in whole animals. Having found that the cellular structure of the cochlea was intact in *Celf4*^±^ mice, we next moved on to examine functions of ribbon synapses in inner hair cells (IHCs) in the cochlea. We first performed patch-clamp recording in IHCs, applied voltage ramps and recorded I_Ca_ (Fig. 4A). We performed curve fitting on traces of I_Ca_, yielding three parameters including the I_Ca_ peak, V_half_ and slope *k*, none of which was significantly changed (WT: I_Ca_ = 207 ± 30.2 pA, V_half_ = −17.9 ± 4.99 mV, slope *k* = 8.55 ± 0.959 mV, n = 22 cells; *Celf4*^±^: I_Ca_ = 190 ± 42.4 pA, V_half_ = −17.6 ± 5.18 mV, slope *k* = 8.53 ± 1.02 mV, n = 41 cells; I_Ca_: two-tailed Mann–Whitney *U* test, p = 0.108, V_half_: two-tailed unpaired *t*-test, t_(df = 61)_ = 0.195, p = 0.846, slope *k*: two-tailed unpaired *t*-test, t_(df = 61)_ = 0.0812, p = 0.936, Fig. 4B). We then performed whole-cell capacitance measurement to assess exocytosis from IHCs. As shown in Fig. 4C, in response to step depolarization of 50 (left) and 500 ms (right), IHCs produced a I_Ca_ and a capacitance jump (ΔC_m_). We found no significant difference in ΔC_m_ (50 ms duration: 14.5 ± 13.1 fF for WT IHCs, n = 22 cells; 15.3 ± 5.55 fF for *Celf4*^±^ IHCs, n = 41 cells; 500 ms duration: 70.5 ± 33.3 fF for WT IHCs, n = 22 cells; 79.8 ± 66.8 fF for *Celf4*^±^ IHCs, n = 41 cells; two-way ANOVA: genotype effect: F_(1, 40)_ = 1.93, p = 0.173; interval effect: F_(7, 280)_ = 71.4, p < 0.0001; interaction: F_(7, 74)_ = 0.372, p = 0.916, Fig. 4D). To compare kinetics of exocytosis, we performed curve fitting (Fig. 4E) on data in Fig. 4D, yielding an estimate of the readily releasable pool (RRP) of synaptic vesicles, sustained release rate (SRR) of synaptic vesicles and the time constant for depleting RRP. Once again, none of these three parameters on exocytosis kinetics was significantly changed in *Celf4*^±^ IHCs (WT: RRP = 595 ± 195 SVs, SRR = 2638 ± 1705 SVs/s, RRP depletion time constant = 34.2 ± 34.3 ms, n = 18 cells; *Celf4*^±^: RRP = 533 ± 225 SVs, SRR = 2489 ± 922 SVs/s, RRP depletion time constant = 34.2 ± 31.53 ms, n = 29 cells; RRP: two-tailed unpaired *t*-test, t_(df = 45)_ = 0.959, p = 0.343, SRR: two-tailed Mann–Whitney *U* test, p = 0.641, RRP depletion time constant: two-tailed Mann–Whitney *U* test, p = 0.905, Fig. 4F). Taken together, these results suggested that function of ribbon synapses in IHCs were intact in *Celf4*^±^ cochleae.

### Synaptic Vesicle Release was Subtly Reduced at the Endbulb of Held Synapse

**Fig. 5:**
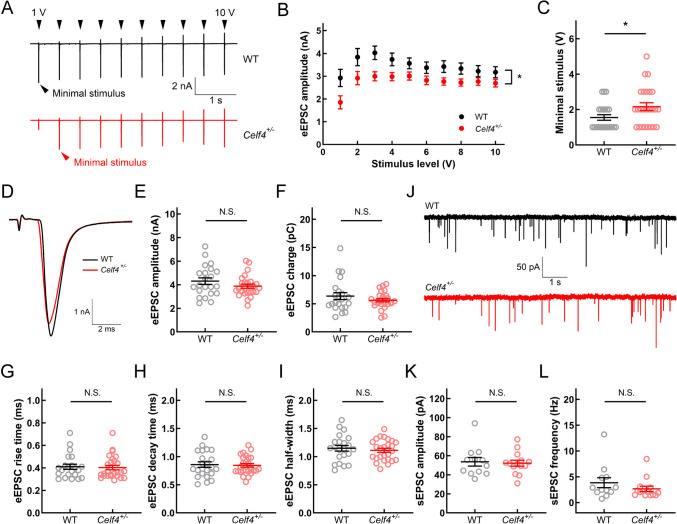
Release of synaptic vesicles was subtly but significantly reduced at the endbulb of Held synapse in the cochlear nucleus in *Celf4*^±^ mice. **A** Representative trace of evoked EPSCs (eEPSCs) recorded from a WT and a *Celf4*^±^ bushy cell while the auditory nerve was stimulated with voltage pulses at increasing levels (1–10 V, 1 V increment). The minimal stimulus is defined as the voltage level at which a large and consistent eEPSC was obtained (arrowhead). **B** Plot of eEPSC amplitude against stimulation level. Note that the eEPSC amplitude was subtly but significantly reduced in *Celf4*^±^ bushy cells. **C** Pooled data for the minimal stimulus in WT and *Celf4*^±^ bushy cells, showing that stronger stimulation was needed to induce large and consistent eEPSCs in *Celf4*^±^ bushy cells. **D** Typical eEPSCs recorded from a WT and *Celf4*^±^ bushy cell pair. Note that the stimulation artifact is clearly distinguishable from the synaptic current. **E**–**I** Parameters of eEPSCs, including the amplitude (**E**), charge (**F**), rise time (**G**), decay time (**H**) and half-width (**I**), none of which was changed significantly in *Celf4*^±^ bushy cells. However, both the amplitude (**E**) and charge (**F**) exhibited a trend of decrease, consistent with data in B. **J** Representative recordings of spontaneous EPSCs (sEPSCs) in a WT and *Celf4*^±^ bushy cell pair. **K** and **L** Amplitude (**K**) and frequency (**L**) of sEPSCs for WT and *Celf4*^±^ bushy cells, neither of which was changed in *Celf4*^±^ bushy cells. For eEPSCs, data were collected from 5 WT mice (n = 22 cells) and 5 *Celf4*^±^ mice (n = 26 cells). For sEPSCs, data were collected from 4 WT mice (n = 12 cells) and 4 *Celf4*^±^ mice (n = 14 cells)

After entering the brainstem, the central axon of SGNs bifurcates, and the ascending branch makes giant synaptic contact on bushy cells in AVCN, which is often referred to as the endbulb of Held synapse (Yu and Goodrich 2014). This giant synapse contains a large number of release sites, allowing auditory signals to be transmitted at high rates and with high precision (Xie 2016). In order to examine if and how the endbulb of Held synapse was affected in *Celf4*^±^ mice, we prepared brainstem slices, applied a suction pipette on the auditory nerve stump, and stimulated auditory nerve fibers extracellularly with brief voltage pulses of ascending voltages (Fig. 5A). We found that evoked excitatory postsynaptic currents (eEPSCs) were significantly smaller in *Celf4*^±^ mice (For 3 V stimulation: 4.03 ± 1.38 nA in WT mice, n = 22 cells; 3.00 ± 0.996 nA in *Celf4*^±^ mice, n = 25 cells; two-way ANOVA: genotype effect: F_(1, 24)_ = 6.63, p = 0.0166; interval effect: F_(9, 216)_ = 8.24, p < 0.0001; interaction: F_(9, 186)_ = 1.32, p = 0.230, Fig. 5B). Moreover, to evoke large eEPSCs (> 2 nA) with constant amplitude, a larger minimal stimulation (1.55 ± 0.739 V for WT mice, n = 22 cells; 2.16 ± 1.11 V for *Celf4*^±^ mice, n = 25 cells; two-tailed Mann–Whitney *U* test, p = 0.0425, Fig. 5C) was required for *Celf4*^±^ mice. However, when we pooled together the half activation voltage for individual bushy cells (V_half_), we found no significant change in *Celf4*^±^ mice, indicating that the larger minimal stimulus required for *Celf4*^±^ mice might not be due to reduced axonal excitability but merely an embodiment of reduced amplitudes in eEPSCs (1.63 ± 1.18 V for WT mice, n = 25 cells; 1.74 ± 1.42 V for *Celf4*^±^ mice, n = 15 cells; two-tailed Mann–Whitney *U* test, p = 0.841, Supplementary Fig. 6). With the minimal stimulation, the amplitude and charge of eEPSCs both exhibited a trend of reduction, but neither of the differences was significant statistically (Fig. 5D–F). Furthermore, the kinetics of eEPSCs was not changed either (Fig. 5G–I). Lastly, we recorded spontaneous EPSC (sEPSCs) in bushy cells of WT and *Celf4*^±^ mice (Fig. 5J), and we found neither the amplitude nor the frequency of sEPSCs was significantly changed in *Celf4*^±^ mice (For amplitude: 53.6 ± 15.6 pA in WT mice, n = 12 cells; 52.2 ± 11.7 pA in *Celf4*^±^ mice, n = 14 cells; For frequency: 3.85 ± 3.37 Hz in WT mice, n = 12 cells; 2.66 ± 1.93 Hz in *Celf4*^±^ mice, n = 14 cells; amplitude: two-tailed Mann–Whitney *U* test, p = 0.781, frequency: two-tailed Mann–Whitney *U* test, p = 0.274, Fig. 5K and L), suggesting that the quantal size at the endbulb of Held synapse remained unchanged.

**Fig. 6:**
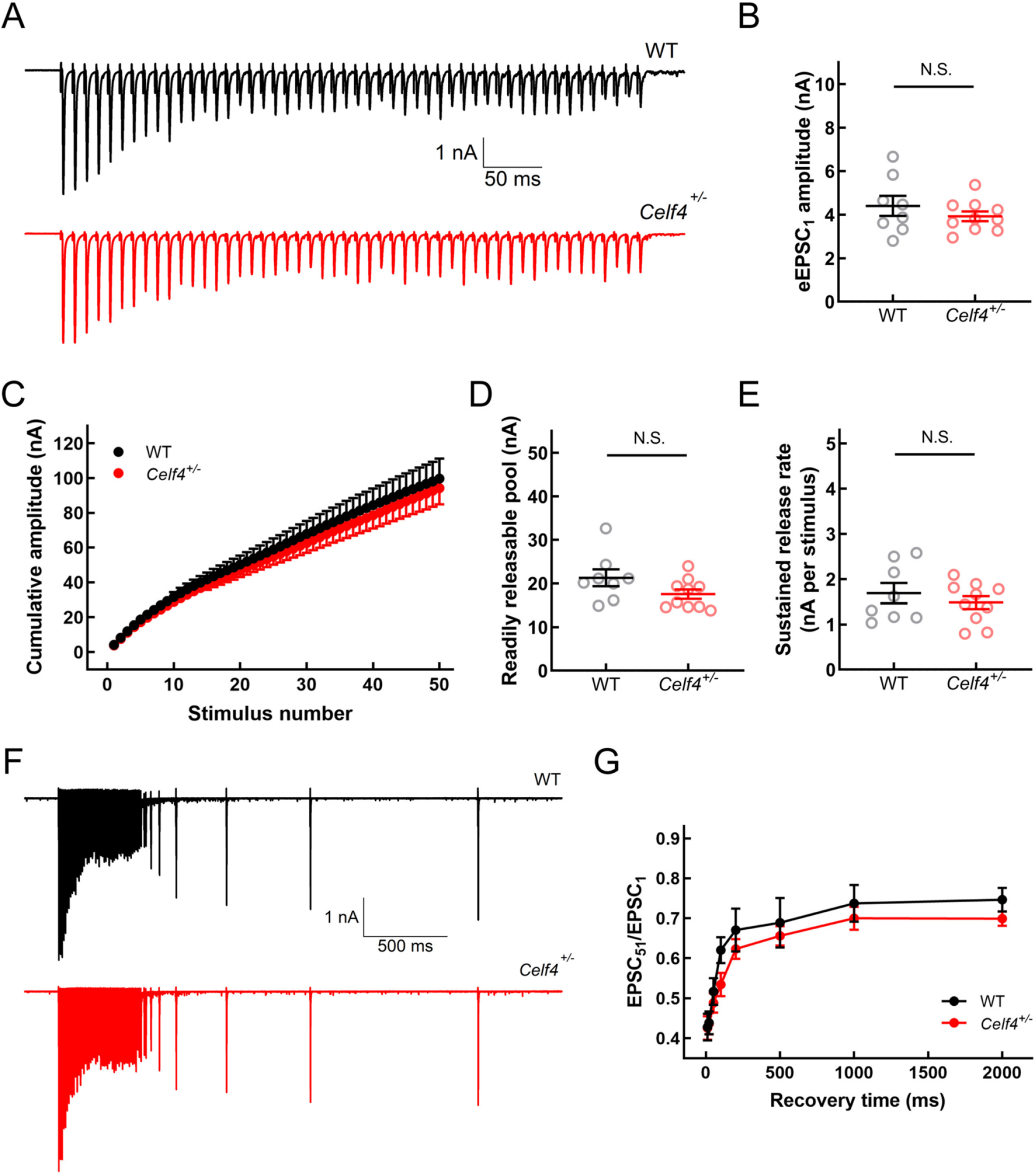
Recycling of synaptic vesicles at the endbulb of Held synapse remained intact in *Celf4*^±^ mice. **A** Representative EPSC recordings in response to 50 stimulations delivered at 100 Hz in a WT and *Celf4*^±^ bushy cell pair. **B** Summary of the average amplitude for EPSC_1_, which was not significantly changed in *Celf4*^±^ mice. **C** Summary of the cumulative EPSC amplitude plotted against the stimulus number, showing that the release of synaptic vesicles remained unchanged in *Celf4*^±^ mice. **D** and **E** Pooled data for the readily releasable pool of synaptic vesicles (**D**) and the sustained release rate of synaptic vesicles (**E**), calculated from data in C (see Material and Methods), neither of which was changed in *Celf4*^±^ mice. **F** Typical EPSC recordings in response to 50 stimulations at 100 Hz, followed by a 51st stimulation delivered at varied intervals. **G** Summary of the amplitude ratio of EPSC_51_/EPSC_1_ derived from data shown in F from multiple cells, showing that the recycling of synaptic vesicles was comparable between WT and *Celf4*^±^ mice. Data were collected from 4 WT (n = 8 cells) and 3 *Celf4*^±^ mice (n = 10 cells)

We next examined release of synaptic vesicles at the endbulb of Held synapse with 50 stimulations delivered at 100 Hz. In WT mice, we observed synaptic depression as the amplitude of eEPSCs decreased and reached a steady state, an indication of depleting the readily releasable pool (RRP) of synaptic vesicles (Fig. 6A). The release of synaptic vesicles behaved similarly in *Celf4*^±^ mice, and the amplitude of first eEPSC exhibited a trend of reduction but the difference was not significant statistically (Fig. 6B). We then calculated the cumulative amplitudes of evoked EPSCs over time, and we found that once again the release of synaptic vesicles exhibited a trend of reduction in *Celf4*^±^ mice, but the difference was not significant statistically (99.8 ± 32.4 nA for WT, n = 8 cells; 94.2 ± 29.5 nA for *Celf4*^±^, n = 10 cells; two-way ANOVA, genotype effect: F_(1, 9)_ = 0.289, p = 0.604, Fig. 6C). Upon further analysis, we found that both RRP and the sustained release rate (SRR) of synaptic vesicles exhibited a trend of reduction in *Celf4*^±^ mice, but the difference was not significant statistically (WT: RRP = 21.3 ± 5.46 nA, SRR = 1.69 ± 0.633 nA per stimulus, n = 8 cells; *Celf4*^±^: RRP = 17.5 ± 3.40 nA, SRR = 1.48 ± 0.447 nA per stimulus, n = 10 cells; RRP: two-tailed unpaired *t*-test, t_(df = 16)_ = 1.78, p = 0.0936, SRR: two-tailed unpaired *t*-test, t_(df = 16)_ = 0.808, p = 0.431, Fig. 6D and E). We next divided the quantal content with RRP, and we found no significant change in the release probability (0.209 ± 0.0478 for WT mice, 0.226 ± 0.0319 for *Celf4*^±^ mice, two-tailed unpaired *t*-test, t_(df = 16)_ = 0.894, p = 0.384). Lastly, in order to probe the recovery of RRP, we applied a 51st stimulation after 50 stimulations with varied intervals (Fig. 6F). In Fig. 6G, we plotted the ratio of EPSC_51_/EPSC_1_ against the interval, and we found no difference in the recovery of RRP between WT and *Celf4*^±^ mice. Taken Figs. 5 and 6 together, these results indicated the release of synaptic vesicles was subtly reduced at the endbulb of Held synapse in *Celf4*^±^ mice.

### Excitability of Bushy Cells in the Cochlear Nucleus was Significantly Decreased in Celf4^±^ Mice

**Fig. 7:**
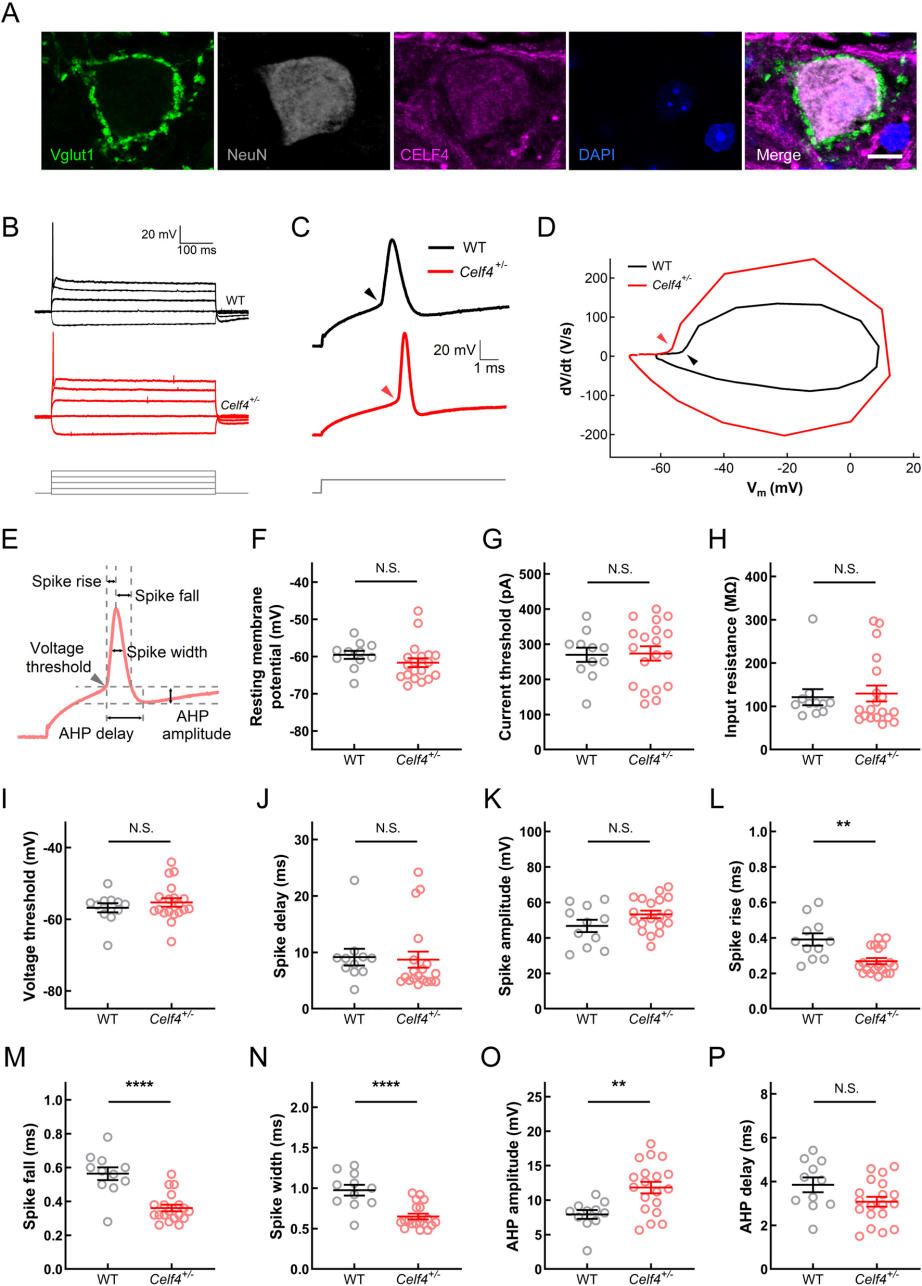
Kinetics of spikes were significantly faster in *Celf4*^±^ bushy cells. **A** Representative images of a WT bushy cell, quadruple immunolabeled for Vglut1 (synaptic puncta, green), NeuN (neurons, gray), CELF4 (targeted protein, magenta) and DAPI (nuclei, blue). Scale bar: 10 μm. **B** Representative voltage responses to step current injections in a WT and *Celf4*^±^ bushy cell pair. Between stimulations, all cells were held at a small negative current to maintain the cell at approximately −90 mV. **C** Spikes elicited by the minimal current injection that was sufficient to trigger a spike, in a WT and *Celf4*^±^ bushy cell pair. The amplitude of the current injected was defined as the current threshold (see pooled data in G). **D** Plots of dV/dt versus instantaneous membrane potential, derived from the traces in C, showing significant change of spike kinetics in *Celf4*^±^ bushy cells. Arrowheads indicate the onset of spikes. **E** Schematic diagram illustrating definitions of parameters for spike kinetics. **F**–**P** Summary of intrinsic properties in WT and *Celf4*^±^ bushy cells. Note that spikes in *Celf4*^±^ bushy cells exhibited faster rise and fall (**L** and **M**), narrower width (**N**), and larger afterhyperpolarization (AHP, **O**). Data were collected from 3 WT (n = 11 cells) and 3 *Celf4*^±^ mice (n = 19 cells)

Other than synaptic inputs, intrinsic properties and excitability of neurons also bear tremendous amount of impact on their spiking responses. In order to investigate if and how intrinsic properties and excitability of bushy cells were affected in *Celf4*^±^ mice, we first determined CELF4 was indeed expressed in bushy cells (Fig. 7A) and then performed patch-clamp recording in bushy cells in brainstem slices. To bring all cells to the same starting voltage, we turned on “the gentle switch” in the acquisition software (PatchMaster, Heka), so that cells were held at a small negative current to maintain a membrane potential of approximately −90 mV when we switched from the voltage-clamp mode (cells were held at −90 mV under voltage-clamp) to the current-clamp mode. To measure the resting membrane potential, we first stepped the cell to zero current, and we found no change in *Celf4*^±^ mice (Fig. 7F). We then applied step current injection of increasing amplitudes, and we found that bushy cells always fired a single spike (Fig. 7B–D), consistent with their physiological characteristics published by multiple groups (Wu and Oertel 1984; Xie 2016; Antunes et al. 2020). We took the voltage response with the largest current injection but without triggering a spike and calculated the input resistance, and we found no difference between WT and *Celf4*^±^ mice (Fig. 7H). Lastly, we took the voltage response with the least current injection but triggering a spike and calculated parameters of spikes (Fig. 7E). We found no significant change in the current threshold, the voltage threshold, the spike delay or the spike amplitude (Fig. 7G and I–K). Significantly, we found that the rise time, the fall time and the width of spikes in *Celf4*^±^ bushy cells were shortened (WT: rise time = 0.391 ± 0.115 ms, fall time = 0.564 ± 0.126 ms, spike width = 0.975 ± 0.220 ms, n = 11 cells; *Celf4*^±^: rise time = 0.264 ± 0.0749 ms, fall time = 0.361 ± 0.0842 ms, spike width = 0.650 ± 0.150 ms, n = 19 cells; rise time: two-tailed unpaired *t*-test, t_(df = 28)_ = 3.54, p = 0.0014, fall time: two-tailed unpaired *t*-test, t_(df = 28)_ = 5.29, p < 0.0001, spike width: two-tailed unpaired *t*-test, t_(df = 28)_ = 4.82, p < 0.0001, Fig. 7L–N). Furthermore, the amplitude of the after hyperpolarization (AHP) became significantly larger in *Celf4*^±^ bushy cells (7.93 ± 2.11 mV for WT, n = 11 cells; 11.8 ± 3.65 mV for *Celf4*^±^, n = 19 cells; two-tailed unpaired *t*-test, t_(df = 28)_ = 3.23, p = 0.0032, Fig. 6O), but its delay remained unchanged (3.85 ± 1.11 ms for WT, n = 11 cells; 3.08 ± 0.978 ms for *Celf4*^±^, n = 19 cells; two-tailed unpaired *t*-test, t_(df = 28)_ = 1.98, p = 0.0575, Fig. 7P).

**Fig. 8:**
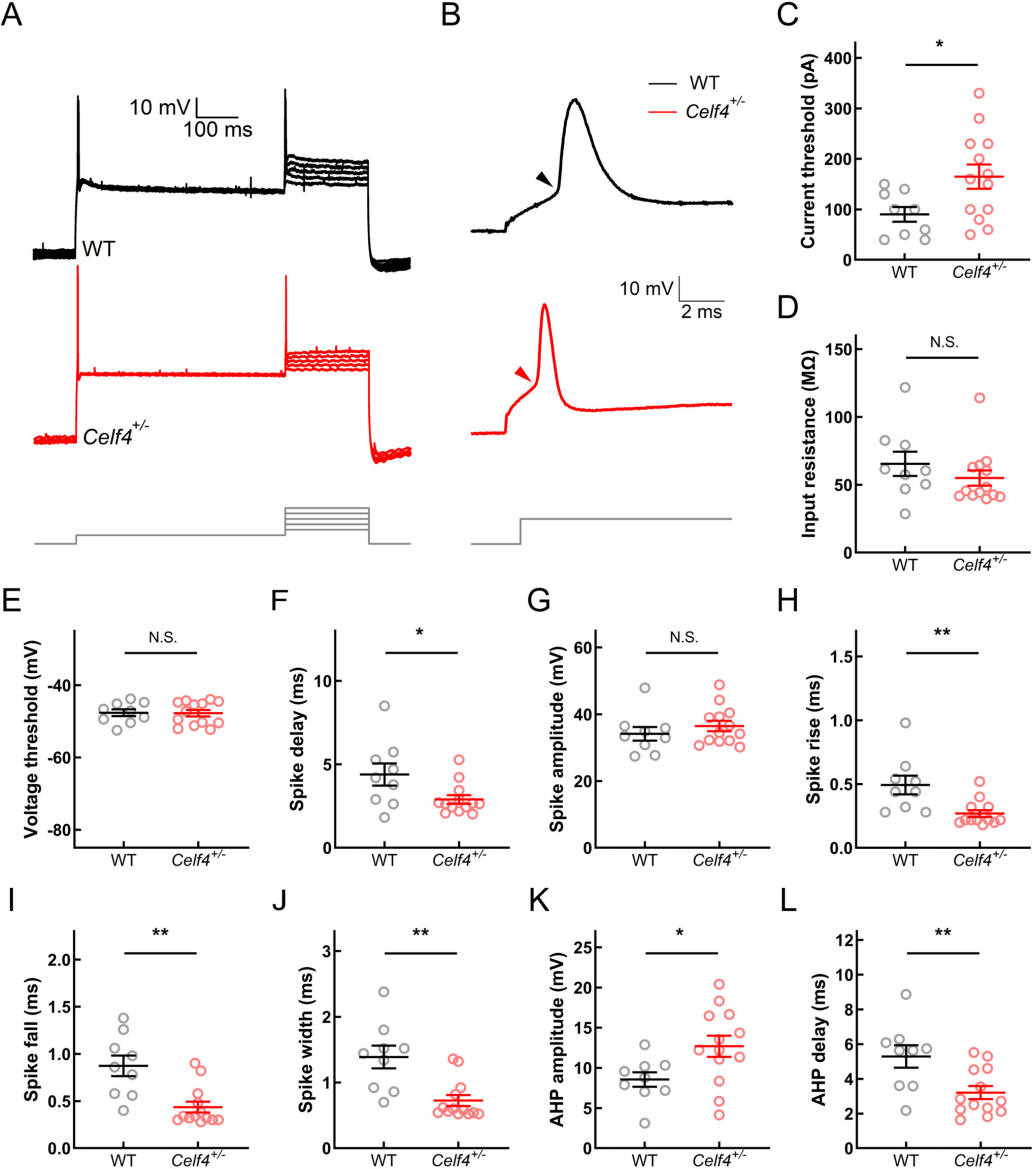
Excitability was significantly decreased in *Celf4*^±^ bushy cells. **A** Representative voltage responses to step current injections, starting from the resting membrane potential. **B** A pair of spikes elicited in a WT and a *Celf4*^±^ bushy cell by injecting the minimal current required to trigger a spike (defined as the current threshold). Pooled current threshold data are shown in C. **C**–**L** Summary of intrinsic properties for WT and *Celf4*^±^ bushy cells. Consistent with the data from the previous figure, spike kinetics were significantly faster (**H**, **I**, **J** and **K**). Different from the data in the previous figure, *Celf4*^±^ bushy cells required a larger current to trigger a spike when compared to WT bushy cells (**C**), indicating decreased excitability. Also different from the data in the previous figure, the spike delay was significantly shorter (**F**), and the AHP delay was also significantly shortened (**L**). Data were collected from 3 WT (n = 9 cells) and 3 *Celf4*^±^ mice (n = 13 cells)

To examine excitability of bushy cells in more physiological conditions, we first stepped the cell to zero current to keep the cell at its resting membrane potential, and then applied step current injection of increasing amplitudes (Fig. 8A and B). We found the resting membrane potential in *Celf4*^±^ bushy cells was more hyperpolarized than their WT counterparts at a pre-pulse of zero current and before the spike (−57.9 ± 3.22 mV for WT, n = 9 cells; −63.1 ± 3.01 mV for *Celf4*^±^, n = 13 cells; two-tailed unpaired *t*-test, t_(df = 20)_ = 3.87, p = 0.001). Consistent with Fig. 7, we found that the input resistance, the voltage threshold and the spike amplitude remained unchanged in *Celf4*^±^ bushy cells (Fig. 8D, E and G), and that the spike rise, the spike fall, the spike width and the AHP amplitude were similarly changed in *Celf4*^±^ bushy cells (Fig. 8H–K). Different from Fig. 7, however, we found that the current threshold was significantly increased (90.0 ± 43.9 pA for WT, n = 9 cells; 165 ± 86.8 pA for *Celf4*^±^, n = 13 cells; two-tailed unpaired *t*-test, t_(df = 20)_ = 2.37, p = 0.0282, Fig. 8C), indicating reduced excitability in *Celf4*^±^ bushy cells. Also different from Fig. 7, we found that both the spike delay and the AHP delay were significantly shortened in *Celf4*^±^ bushy cells (WT: spike delay = 4.39 ± 2.00 ms, AHP delay = 5.29 ± 1.95 ms, n = 9 cells; *Celf4*^±^: spike delay = 2.90 ± 0.926 ms, AHP delay = 3.21 ± 1.34 ms, n = 13 cells; spike delay: two-tailed unpaired *t*-test, t_(df = 20)_ = 2.37, p = 0.0277, AHP delay: two-tailed unpaired *t*-test, t_(df = 20)_ = 2.98, p = 0.0074, Fig. 8F and L).

**Fig. 9:**
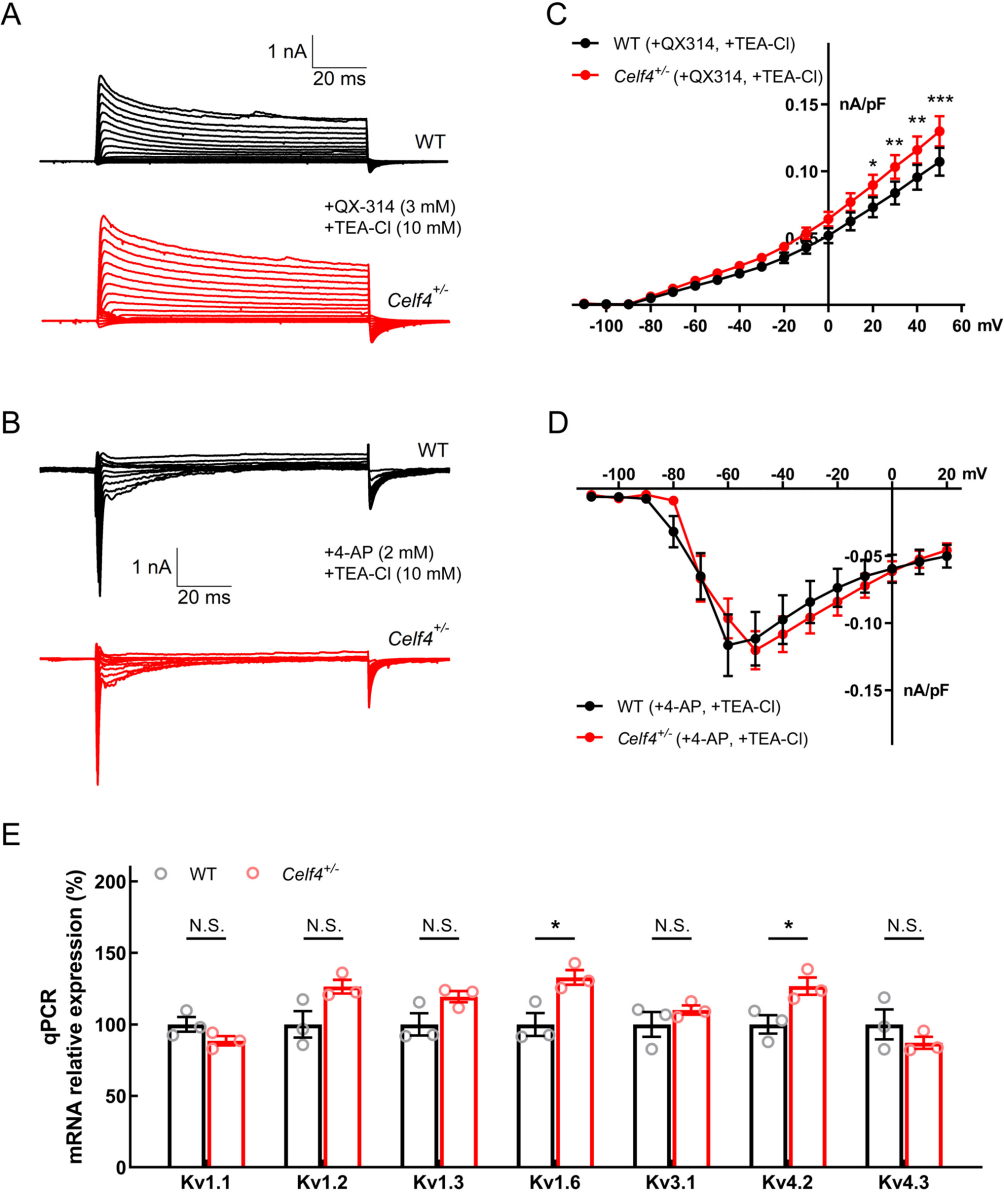
A-type potassium current was significantly increased in *Celf4*^±^ bushy cells. **A** and **B** Representative recordings of A-type potassium current (I_A_, **A**) and sodium current (I_Na_, **B**) in a WT and a *Celf4*^±^ bushy cell. Note that we included QX-314 in the internal solution and TEA-Cl in the external solution to isolate I_A_ currents, and we used a Cs⁺-based internal solution and included TEA-Cl and 4-AP in the external solution to isolate I_Na_ currents. **C** and **D** Current–Voltage (I–V) curves for I_A_ (**C**) and I_Na_ (**D**), showing that while I_Na_ remained unchanged, I_A_ was significantly increased in *Celf4*^±^ bushy cells. **E** mRNA relative expression of Kv1.1, Kv1.2, Kv1.3, Kv1.6, Kv3.1, Kv4.2 and Kv4.3, showing that Kv1.6 and Kv4.2 were significantly upregulated in *Celf4*^±^ mice. For I_A_, data were collected from 4 WT (n = 13 cells) and 4 *Celf4*^±^ mice (n = 15 cells). For I_Na_, data were collected from 3 WT (n = 11 cells) and 3 *Celf4*^±^ mice (n = 24 cells). For qPCR, data were collected from 3 WT and 3 *Celf4*^±^ mice

Given the faster spike kinetics observed in *Celf4*^±^ bushy cells, we hypothesized that voltage gated K^+^ and Na^+^ currents in these neurons were likely to be affected by *Celf4*^±^ haploinsufficiency. We therefore applied voltage steps under voltage-clamp and recorded voltage-gated currents in bushy cells. As shown in Fig. 9A and B, A-type potassium current (I_A_) was isolated by including QX-314 in the internal solution and TEA-Cl in the external solution, and voltage-gated Na^+^ current (I_Na_) was isolated by using a Cs⁺-based internal solution and including TEA-Cl and 4-AP in the external solution. We found that while the amplitude of I_Na_ remained unchanged in *Celf4*^±^ bushy cells (at −50 mV: −0.112 ± 0.0661 nA/pF in WT bushy cells, n = 11 cells; −0.120 ± 0.0699 nA/pF in *Celf4*^±^ bushy cells, n = 24 cells; two-way ANOVA: genotype effect: F_(1, 25)_ = 0.00639, p = 0.937; interval effect: F_(13, 325)_ = 31.2, p < 0.0001; interaction: F_(13, 87)_ = 0.757, p = 0.702, Fig. 9D), the amplitude of I_A_ was significantly increased at multiple membrane voltages (at 50 mV: 0.107 ± 0.0377 nA/pF in WT bushy cells, n = 13 cells; 0.130 ± 0.0438 nA/pF in *Celf4*^±^ bushy cells, n = 15 cells; two-way ANOVA: genotype effect: F_(1, 15)_ = 5.50, p = 0.0333; interval effect: F_(16, 240)_ = 135, p < 0.0001; interaction: F_(16, 172)_ = 4.60, p < 0.0001, Fig. 9C). To investigate the molecular basis of the altered excitability in *Celf4*^±^ bushy cells, we calculated mRNA relative expression in AVCN tissue from *Celf4*^±^ mice and their WT littermates. We analyzed the expression of key voltage-gated potassium subunits (Kv1.1, Kv1.2, Kv1.3, Kv1.6, Kv3.1, Kv4.2, Kv4.3) known to shape bushy cell firing properties. We found that the relative expression of Kv1.6 and Kv4.2 was significantly upregulated in *Celf4*^±^ mice (For Kv1.6: two-tailed unpaired *t*-test, t_(df = 4)_ = 3.46, p = 0.0259; For Kv4.2: two-tailed unpaired *t*-test, t_(df = 4)_ = 3.02, p = 0.0392, Fig. 9E), while the transcript levels of Kv1.1, Kv1.2, Kv1.3, Kv3.1, and Kv4.3 remained unchanged between genotypes. Taken together, we found increased I_A_ and mRNA expression of Kv1.6 and Kv4.2 in *Celf4*^±^ bushy cells, suggesting that *Celf4* might regulate I_A_ by upregulating Kv1.6 and Kv4.2.

## Discussion

Haploinsufficiency of CELF4 causes 18q12.2 deletion syndrome characterized by multiple neurodevelopmental deficits including anxiety, ADHD, intellectual disability, and speech and language delay. Although CELF4 has been shown to regulate excitability and synaptic transmission in the central nervous system, whether CELF4 has same effect on neurons and synapses in the auditory pathways is still unknown. Here, we generated a *Celf4*^±^ mouse line to simulate clinical human phenotypes and investigated functional changes in the first two synapses in the auditory pathways. We found that hearing functions remained largely intact in *Celf4*^±^ mice in vivo, and the unchanged I_Ca_ and exocytosis suggested the function of ribbon synapses was also intact in *Celf4*^±^ IHCs. Furthermore, synaptic vesicle release was subtly reduced at the endbulb of Held synapse and the excitability of postsynaptic bushy cells was decreased. Lastly, we found that *Celf4*^±^ bushy cells fired spikes with faster kinetics, likely caused by larger I_A_ current and upregulated voltage-gated potassium channels found in these cells.

### Frequency and Age-Dependent Deficits in Central Auditory Processing

Our in vivo auditory assessments revealed a nuanced and evolving phenotype in *Celf4*^±^ mice that was distinct from canonical hearing loss. The preservation of DPOAE and ABR thresholds across ages indicated that OHC function and cochlear sensitivity remained intact (Figs. 2B, SA). Instead, the observed frequency-specific alterations in ABR amplitudes and latencies pointed to a deficit in neural encoding and synaptic transmission within the central auditory processing.

The most prominent finding in one-month-old *Celf4*^±^ mice was a selective impairment at 4 kHz, characterized by a reduced Wave II amplitude, and prolonged latencies of Wave II and IV at low intensities (Figs. 2D, 2F and S1C). This pattern suggested synaptic efficacy deficit at the endbulb of Held synapse, which is believed to be the primary source of activity for Wave II, and a subsequent delay in downstream brainstem processing (Moller 1981; Moller and Jannetta 1981; Moller et al. 1981; Spire et al. 1982). This was highly consistent with our cellular electrophysiological data showing reduced synaptic strength at this very synapse. The frequency specificity of 4 kHz might arise from the heterogeneous expression of CELF4 within SGNs along the cochlear axis. Transcriptomic data suggested CELF4 expression is dynamic and non-uniform across SGNs (Li et al. 2020), making it plausible that SGNs innervating specific frequency regions exhibited a higher dependency on CELF4 for synaptic maintenance, rendering them more vulnerable to its haploinsufficiency.

Intriguingly, the phenotype underwent an age-dependent shift in 10-month-old mice, where the primary abnormality emerged as shortened Wave II and III latencies at 8 kHz (Supplementary Fig. 3D). This could reflect a compensatory neural plasticity or a pathological remodeling of the auditory brainstem circuits over time (Rumschlag et al. 2022). A chronic reduction in synaptic drive from the auditory nerve might trigger homeostatic increases in postsynaptic excitability or alterations in inhibitory circuitry within the downstream brainstem, ultimately accelerating signal transmission (Mafi et al. 2022). Such latency shifts without threshold elevation are one of a hallmark of central auditory processing disorders (Klug et al. 2000; Strelcyk et al. 2009). Collectively, CELF4 haploinsufficiency likely disrupted the precise protein homeostasis required for optimal synaptic strength and firing fidelity in a subset of SGNs and their central targets, and its deficiency might cause frequency and age-dependent alterations in sound encoding rather than pervasive hearing loss.

### Subtly Reduced Synaptic Transmission at the Endbulb of Held Synapses

Our patch-clamp recordings in bushy cells in vitro revealed a subtle but significant reduction in synaptic strength at the endbulb of Held synapse in *Celf4*^±^ mice. This was evidenced by the smaller amplitude of eEPSCs under stronger stimulation (Fig. 5B) and the requirement for stronger minimal stimulation to elicit large and consistent eEPSCs (Fig. 5C). These findings suggested that *Celf4* haploinsufficiency caused a decrease in the efficacy of synaptic transmission at this critical first central auditory synapse.

The reduction in synaptic strength could arise from several potential mechanisms. Because we found no significant change in the amplitude or rate of quantal EPSCs (Fig. 5K and L), the reduced synaptic strength is likely caused by presynaptic mechanisms, including a decrease in the number of release sites. Although RRP and sustained release rate during high-frequency stimulation did not reach statistical significance, both parameters trended toward lower values in *Celf4*^±^ mice (Fig. 6D and E), consistent with a modest presynaptic deficit. The preservation of synaptic vesicle recycling kinetics (Fig. 6G) suggests that the basic machinery for vesicle recruitment and recovery was intact, and that the defect likely lied in the efficiency of release. Overall. the subtlety of the synaptic transmission deficit might explain why hearing thresholds and basic auditory function remained largely normal in *Celf4*^±^ mice, while ABR wave II latency and amplitude were altered in vivo (Fig. 2D and F).

### Change in Excitability of Bushy Cells

The haploinsufficiency of CELF4 induced a profound and multifaceted change in the intrinsic properties of bushy cells, including decreased neuronal excitability and accelerated action potential kinetics. When stimulated from their resting membrane potential, these cells required a significantly larger injected current to elicit an action potential (Fig. 8C). This indicated that the cells were less responsive to depolarizing inputs, a deficit that could directly dampen the transmission fidelity of auditory information. As for the mechanisms for this decreased excitability in bushy cells, we can rule out the input resistance and the voltage threshold, because neither of them was changed significantly in two independent sets of experiments (Figs. 7H, I, 8D, E). The resting membrane potential was not changed, either, but there is a trend of hyperpolarization (Fig. 7F), likely causing decreased excitability. Furthermore, this change of the resting membrane potential became significant when it was measured at a pre-pulse of zero current and before the spike (Fig. 8A, see statistics in the text). Consistently, we found an increased expression of low voltage-activated Kv1.6 in AVCN (Fig. 9E), likely contributing to hyperpolarization of the resting membrane potential.

Concomitantly, we observed a striking acceleration of action potential kinetics. Spikes in *Celf4*^±^ bushy cells exhibited faster rise and fall times, resulting in a narrower spike width (Figs. 7L–N, 8H–J). Furthermore, following a spike, AHP was significantly larger in amplitude and earlier in time (Figs. 7O, 8K, L). These kinetic changes were electrophysiological signatures often associated with an increased contribution of potassium currents that repolarize the membrane (Fu et al. 2021). Indeed, our voltage-clamp recordings confirmed a significant increase in the I_A_ in *Celf4*^±^ bushy cells (Fig. 9A, C). The upregulation of I_A_ provided a compelling mechanistic explanation for the observed faster spike repolarization and enhanced AHP, as this current activates rapidly upon depolarization and helps to control spike timing and shape (Fu et al. 2021). Supporting this, qPCR analysis revealed a significant upregulation in the mRNA expression of Kv1.6 and Kv4.2 (Fig. 9E), and Kv4.2 is known to mediate rapidly inactivating potassium currents rather than set the resting membrane potential in bushy cells (Pal et al. 2005; Zhang et al. 2025).

### Clinical Relevance and Limitations of the Study

Central auditory processing (CAP) refers to the neural mechanisms responsible for sound localization, discrimination, pattern recognition, and temporal processing within the central nervous system. Deficits in this domain, known as central auditory processing disorder (CAPD), are characterized by impaired neural processing of auditory stimuli rather than higher-order cognitive or language dysfunction (Geffner and Ross-Swain 2019). CAPD frequently co-occurs with various neurodevelopmental conditions, including speech sound disorders, autism spectrum disorder, and specific learning disabilities, whose clinical manifestations show considerable overlap with those of 18q12.2 deletion syndrome resulting from CELF4 haploinsufficiency (Gates et al. 1990; Bamiou et al. 2001; Alcantara et al. 2004; Dyck et al. 2004; Most and Aviner 2009; Most and Michaelis 2012; Iliadou et al. 2015).

In the auditory pathway, bushy cells of the AVCN serve as critical first-stage processors. By the endbulb of Held synapses, they enable precise temporal encoding and rapid transmission of acoustic information to binaural comparison centers in the superior olivary complex, which is essential for sound localization (Smith et al. 1991, 1993). Our findings demonstrated that Celf4 haploinsufficiency altered intrinsic excitability and synaptic function in bushy cells, likely impairing their phase-locking precision and temporal fidelity.

We therefore proposed that dysfunction at this early synaptic relay might represent a contributing neural substrate to CAPD. The abnormal temporal and spectral information transmitted from bushy cells could disrupt higher-order binaural computation and sound localization (Cao and Oertel 2010; van der Heijden et al. 2011), while also compromising the accurate representation of speech cues such as vowel identity (Seikel et al. 2020). Given the established comorbidity between CAPD and neurodevelopmental disorders, the auditory synaptic and cellular deficits reported here might not only help explain the auditory processing-related symptoms observed in 18q12.2 deletion syndrome, but also provide a potential mechanistic link to its broader behavioral and cognitive phenotypes, including social-communication impairments and autism-like features.

While this study provides novel insights into the role of CELF4 in auditory synapse function, several inherent limitations should be acknowledged. Firstly, our investigation was conducted using a constitutive *Celf4*^±^ mouse model. Although this model recapitulates the systemic nature of human 18q12.2 deletion syndrome and provides a uniform, interpretable genetic background for a foundational phenotypic screen, it does not allow for cell-type-specific manipulation. Future studies employing conditional knockout models, particularly using a *Celf4*-lineage-specific Cre driver, will be essential to pinpoint the exact cellular locus of the observed synaptic and excitability deficits. Secondly, technical constraints limited certain direct assessments. Electrophysiological recordings from the soma of SGNs in mature mice remain exceedingly challenging due to progressive calcification of the modiolus, preventing a direct examination of CELF4’s effect on SGN intrinsic properties. Thirdly, the biological complexity of the system introduces interpretive nuance. CELF4 expression within SGNs is dynamic, heterogeneous, and tonotopically varied. Consequently, our whole-tissue analyses represent population averages that may mask critical subpopulation-specific effects. The functional contribution of SGNs that do or do not express CELF4, and how these changes with age and frequency, remains an important question for future research.

## Supplementary Information

Below is the link to the electronic supplementary material.

Amplitudes and latencies for ABR Wave IV were largely unchanged in *Celf4*^±^ mice. (A) Traces of ABRs at 16 kHz in a WT and *Celf4*^±^ mouse pair. (B and C) Amplitudes (B) and latencies (C) for Wave IV in WT (N = 8) and *Celf4*^±^ mice (N = 7) at 4 kHz, 8 kHz, 16 kHz and 32 kHz, plotted against the sound intensity. Note that no significant differences were observed between the two groups, except that the latency at 4 kHz was increased in *Celf4*^±^ mice. Supplementary file1 (TIFF 3025 kb)

Distortion product otoacoustic emissions (DPOAEs) remained intact in *Celf4*^±^ mice. Summary of DPOAE thresholds (A), DPOAE amplitudes at 80 dB SPL (f_2_/f_1_ = 1.2, B), and input–output (IO) functions at 8.7, 17.4 and 34.8 kHz (C) in WT (N = 8) and *Celf4*^±^ mice (N = 8), showing that DPOAEs were comparable between the two groups. Background noise levels are plotted in faint traces. Supplementary file2 (TIFF 1148 kb)

Overall hearing function was largely preserved in 10-month-old *Celf4*^±^ mice. (A) ABR thresholds were comparable between WT (N = 7) and *Celf4*^±^ mice (N = 7), indicating no significant difference was found between the two groups. (B-D) Amplitudes (B and C) and latencies (D) for Wave I to IV in WT and *Celf4*^±^ mice at 4 kHz and 8 kHz, plotted against the sound intensity. Note that latencies of Wave II and Wave III were shortened in *Celf4*^±^ mice at 8 kHz. Numbers above data points represent the number of mice whose ABR thresholds were effectively determined, out of the total number of mice tested. The two numbers are not always the same because for some cases, ABR waveforms are undetectable even with sounds as loud as 90 dB SPL, the maximum sound level the speaker can deliver, so that ABR thresholds cannot be effectively determined. We therefore excluded these cases from statistical analyses, and included only cases with ABR thresholds determined from at least three mice for both genotypes. Supplementary file3 (TIFF 2016 kb)

Counts of HCs in the middle and basal turn remained intact in *Celf4*^±^ cochleae. Representative images of whole-mount cochleae from a WT and a *Celf4*^±^ mouse, quadruple immunolabeled for Myosin7a (hair cells, red), Prestin (outer hair cells, green), Phalloidin (hair bundles, white) and DAPI (nuclei, blue) in the middle (A) and basal turn (B) in WT and *Celf4*^±^ mice. Similar results were obtained in a total of 3 mice for each group. Scale bar: 40 μm. Supplementary file4 (TIFF 11089 kb)

Spiral ganglion neurons in the apical and basal section was intact in *Celf4*^±^ cochleae. Representative images of cochleae transversal section from a WT and a *Celf4*^±^ mouse, immunolabeled for Tuj1 (spiral ganglion neurons, white) in the apical and basal transversal section in WT (A, N = 3) and *Celf4*^±^ (B, N = 3) mice. Scale bars: 50 μm. Supplementary file5 (TIFF 11027 kb)

The excitability of auditory nerve fibers was unchanged in *Celf4*^±^ mice. (A) Plot of normalized eEPSC success rate against stimulation level. (B) Pooled V_half_ for auditory nerve fibers in WT and *Celf4*^±^ mice, obtained by fitting data in A to a Boltzmann function for each cell. No significant difference was found in V_half_ between WT and *Celf4*^±^ mice. Supplementary file6 (TIFF 30514 kb)

Supplementary file7 (XLSX 18 KB)

Supplementary file8 (XLSX 52 KB)

Supplementary file9 (XLSX 15 KB)

## Data Availability

The datasets generated during the current study are available from the corresponding author on reasonable request.
