# Supplementary figures and images for: Celf4 Regulates Excitability of Bushy Cells in the Cochlear Nucleus of the Mouse Brainstem
Source: Cell Mol Neurobiol. 2026 Apr 24;46:101. doi: 10.1007/s10571-026-01732-8 (PMC13243167; doi:10.1007/s10571-026-01732-8)

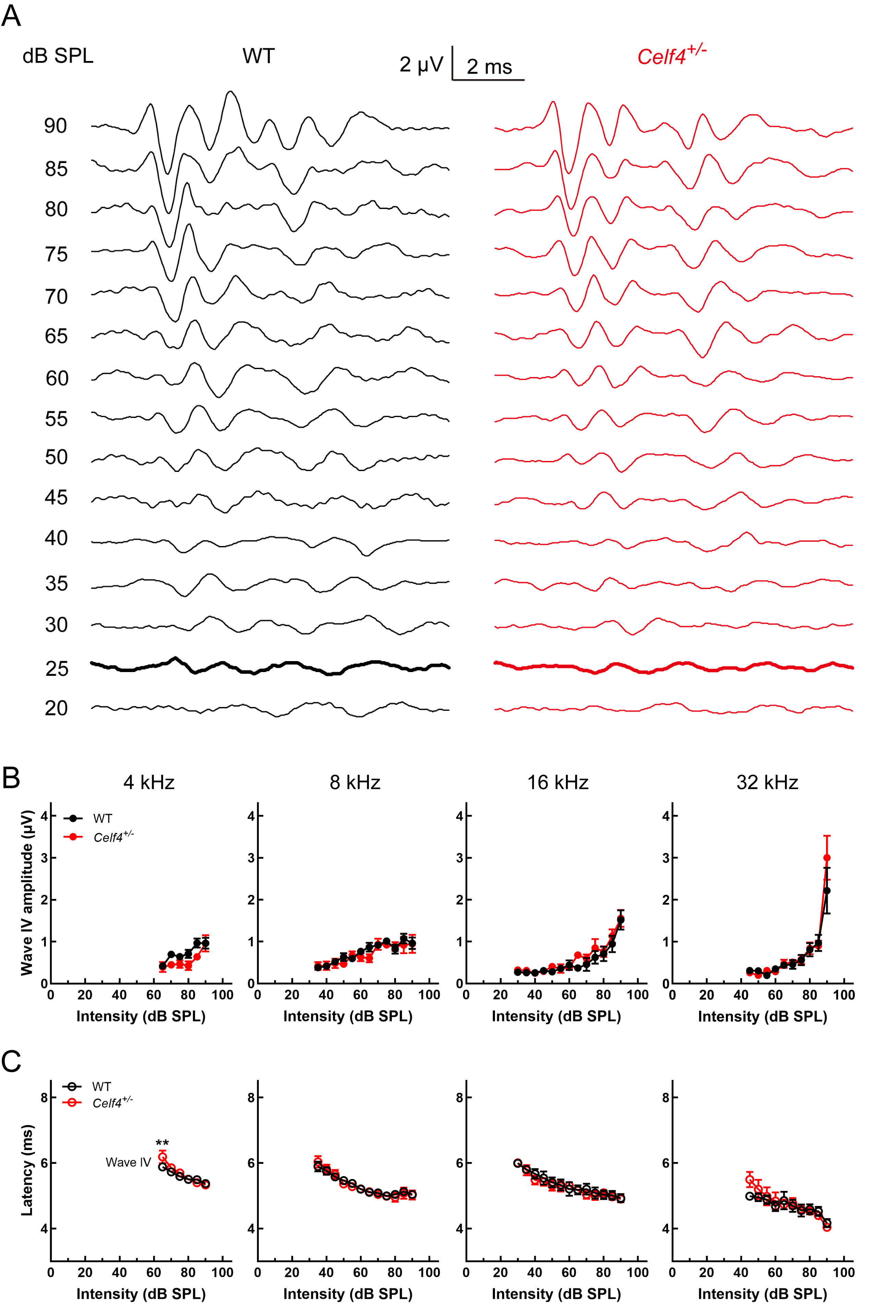

Supplement: Supplementary file 1 — Amplitudes and latencies for ABR Wave IV were largely unchanged in Celf4± mice. (A) Traces of ABRs at 16 kHz in a WT and Celf4± mouse pair. (B and C) Amplitudes (B) and latencies (C) for Wave IV in WT (N = 8) and Celf4± mice (N = 7) at 4 kHz, 8 kHz, 16 kHz and 32 kHz, plotted against the sound intensity. Note that no significant differences were observed between the two groups, except that the latency at 4 kHz was increased in Celf4± mice. Supplementary file1 (TIFF 3025 kb) [file 10571_2026_1732_MOESM1_ESM.tif]

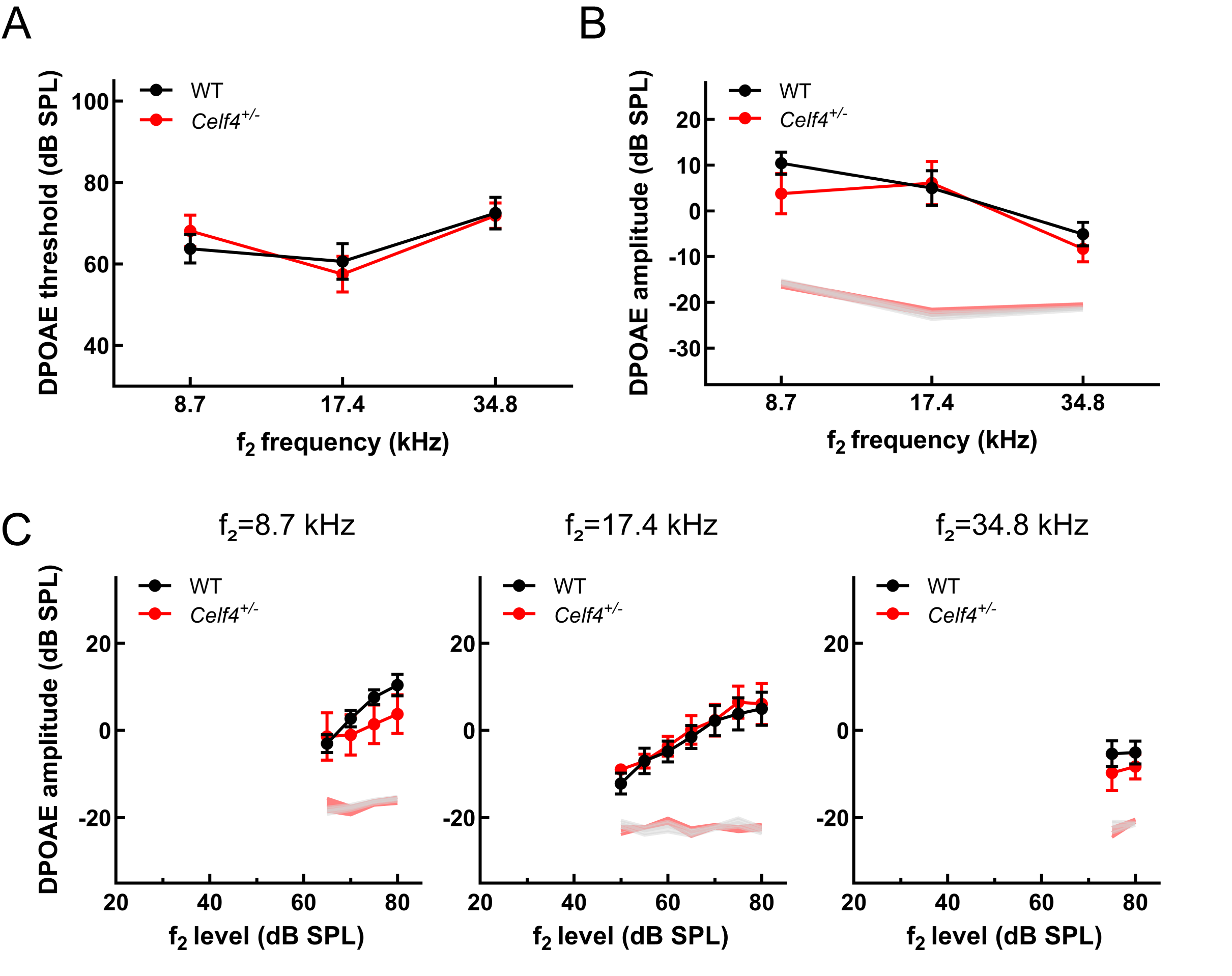

Supplement: Supplementary file 2 — Distortion product otoacoustic emissions (DPOAEs) remained intact in Celf4± mice. Summary of DPOAE thresholds (A), DPOAE amplitudes at 80 dB SPL (f2/f1 = 1.2, B), and input–output (IO) functions at 8.7, 17.4 and 34.8 kHz (C) in WT (N = 8) and Celf4± mice (N = 8), showing that DPOAEs were comparable between the two groups. Background noise levels are plotted in faint traces. Supplementary file2 (TIFF 1148 kb) [file 10571_2026_1732_MOESM2_ESM.tif]

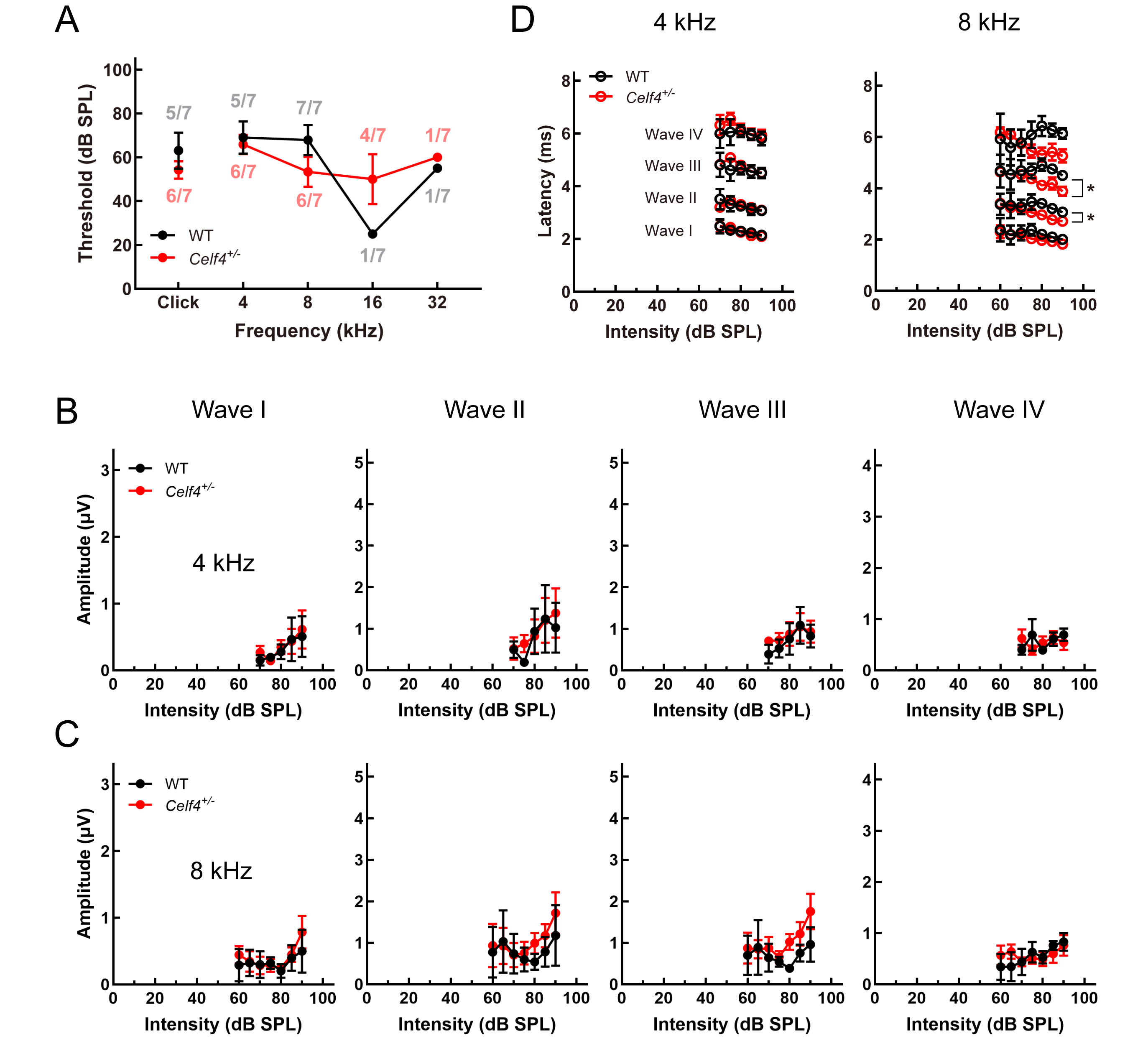

Supplement: Supplementary file 3 — Overall hearing function was largely preserved in 10-month-old Celf4± mice. (A) ABR thresholds were comparable between WT (N = 7) and Celf4± mice (N = 7), indicating no significant difference was found between the two groups. (B-D) Amplitudes (B and C) and latencies (D) for Wave I to IV in WT and Celf4± mice at 4 kHz and 8 kHz, plotted against the sound intensity. Note that latencies of Wave II and Wave III were shortened in Celf4± mice at 8 kHz. Numbers above data points represent the number of mice whose ABR thresholds were effectively determined, out of the total number of mice tested. The two numbers are not always the same because for some cases, ABR waveforms are undetectable even with sounds as loud as 90 dB SPL, the maximum sound level the speaker can deliver, so that ABR thresholds cannot be effectively determined. We therefore excluded these cases from statistical analyses, and included only cases with ABR thresholds determined from at least three mice for both genotypes. Supplementary file3 (TIFF 2016 kb) [file 10571_2026_1732_MOESM3_ESM.tif]

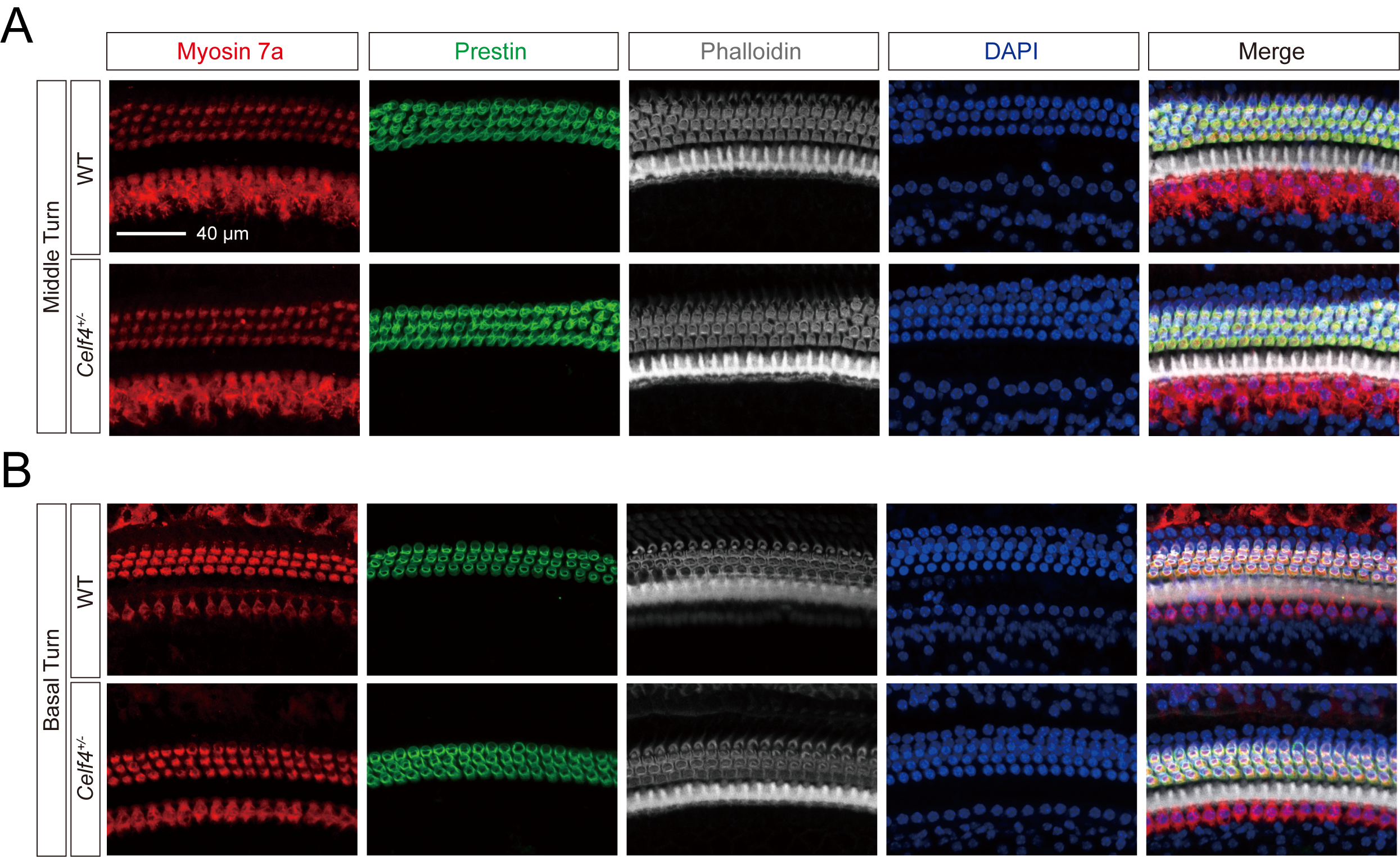

Supplement: Supplementary file 4 — Counts of HCs in the middle and basal turn remained intact in Celf4± cochleae. Representative images of whole-mount cochleae from a WT and a Celf4± mouse, quadruple immunolabeled for Myosin7a (hair cells, red), Prestin (outer hair cells, green), Phalloidin (hair bundles, white) and DAPI (nuclei, blue) in the middle (A) and basal turn (B) in WT and Celf4± mice. Similar results were obtained in a total of 3 mice for each group. Scale bar: 40 μm. Supplementary file4 (TIFF 11089 kb) [file 10571_2026_1732_MOESM4_ESM.tif]

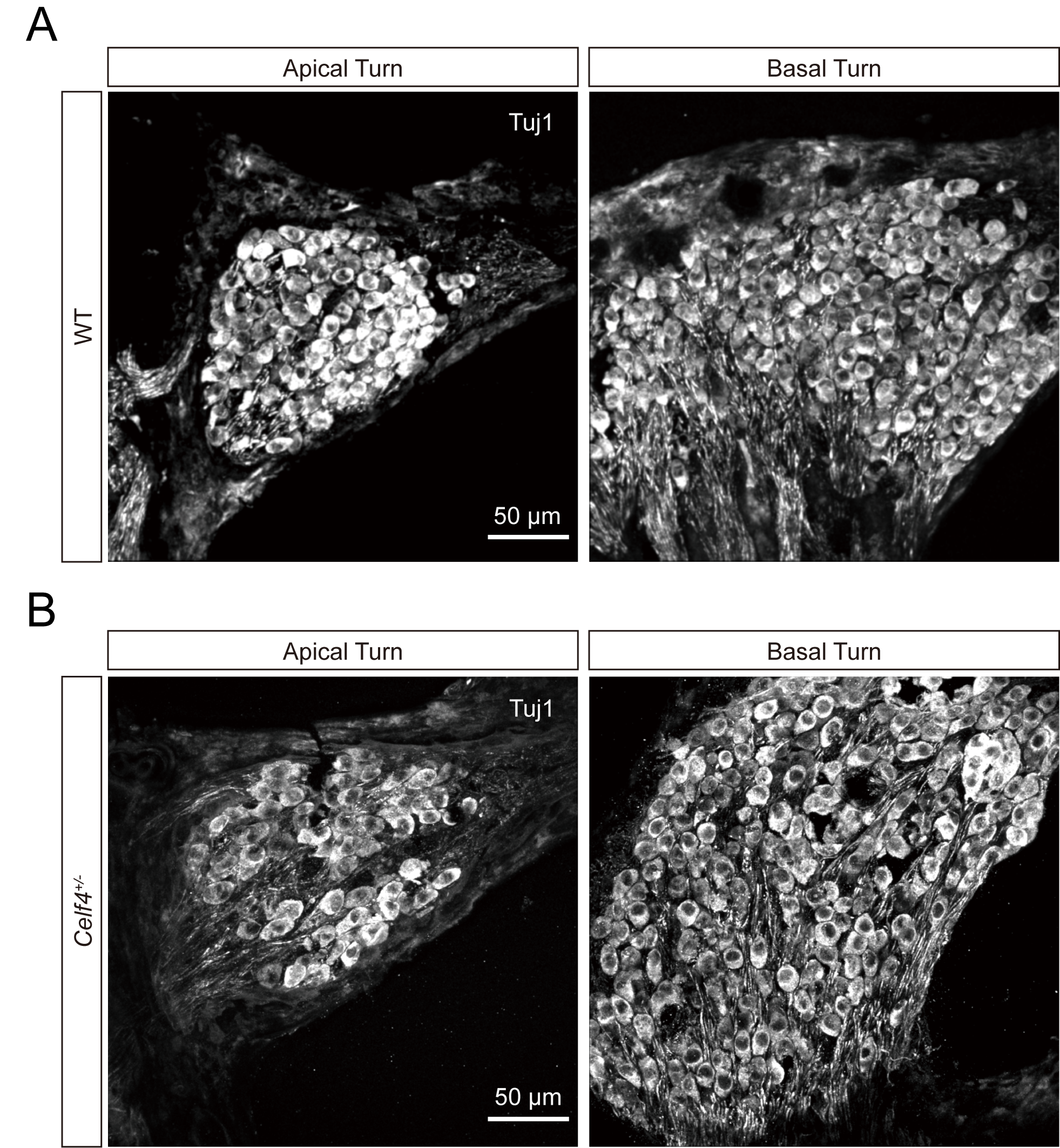

Supplement: Supplementary file 5 — Spiral ganglion neurons in the apical and basal section was intact in Celf4± cochleae. Representative images of cochleae transversal section from a WT and a Celf4± mouse, immunolabeled for Tuj1 (spiral ganglion neurons, white) in the apical and basal transversal section in WT (A, N = 3) and Celf4± (B, N = 3) mice. Scale bars: 50 μm. Supplementary file5 (TIFF 11027 kb) [file 10571_2026_1732_MOESM5_ESM.tif]

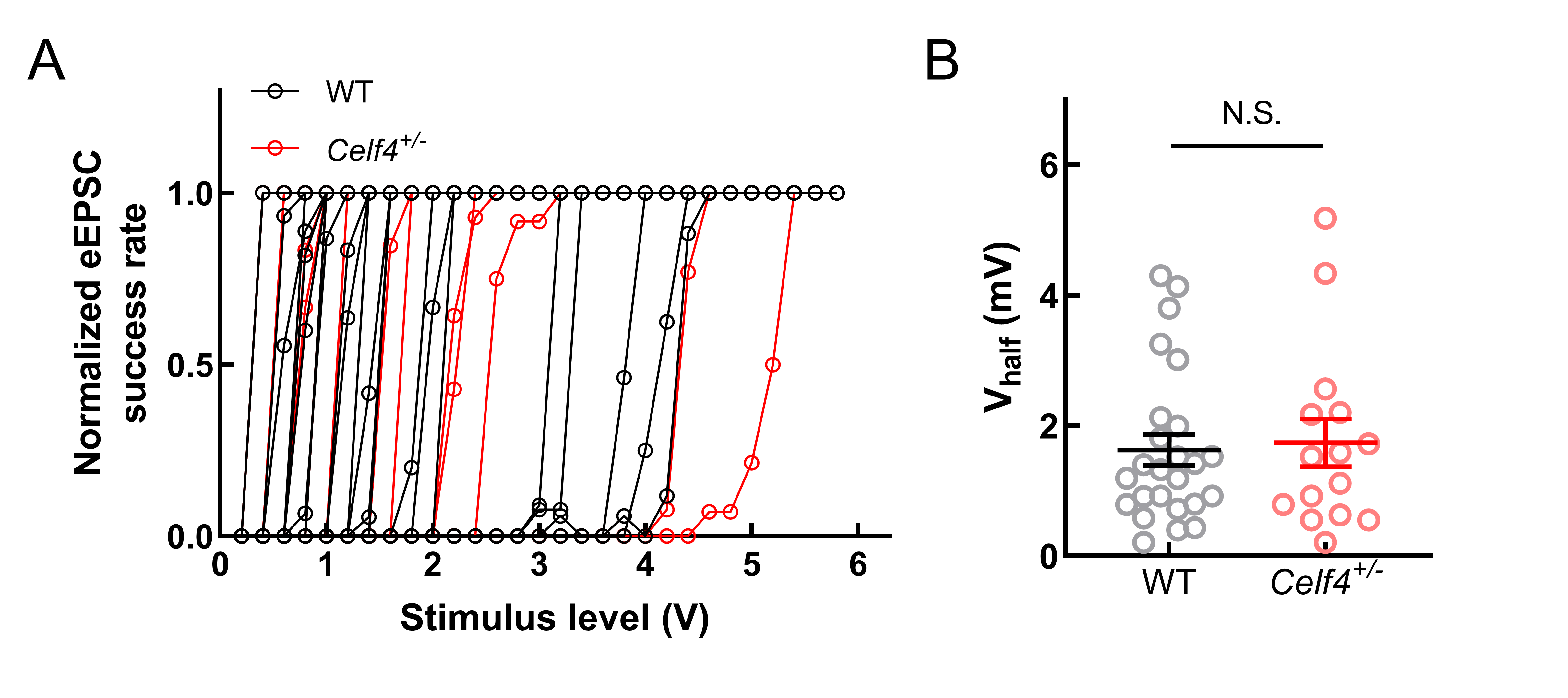

Supplement: Supplementary file 6 — The excitability of auditory nerve fibers was unchanged in Celf4± mice. (A) Plot of normalized eEPSC success rate against stimulation level. (B) Pooled Vhalf for auditory nerve fibers in WT and Celf4± mice, obtained by fitting data in A to a Boltzmann function for each cell. No significant difference was found in Vhalf between WT and Celf4± mice. Supplementary file6 (TIFF 30514 kb) [file 10571_2026_1732_MOESM6_ESM.tif]
